# A clear trade-off exists between the theoretical efficiency and acceptability of dietary changes that improve nutrient adequacy during early pregnancy in French women: Combined data from simulated changes modeling and online assessment survey

**DOI:** 10.1371/journal.pone.0194764

**Published:** 2018-04-11

**Authors:** Clélia M. Bianchi, Jean-François Huneau, Pierre Barbillon, Anne Lluch, Manon Egnell, Hélène Fouillet, Eric O. Verger, François Mariotti

**Affiliations:** 1 UMR PNCA, AgroParisTech, INRA, Université Paris-Saclay, Paris, France; 2 UMR MIA, AgroParisTech, INRA, Université Paris-Saclay, Paris, France; 3 Global Nutrition Department, Danone Nutricia Research, Centre Daniel Carasso, France; 4 NUTRIPASS, IRD, Université Montpellier, SupAgro, Montpellier, France; University Sains Malaysia, MALAYSIA

## Abstract

**Background:**

During pregnancy, the diet of a mother-to-be should be adapted to meet increases in nutrient requirements. We analyzed the theoretical efficiency and acceptability of different types of tailored dietary changes for pregnant women.

**Methods:**

The nutrient adequacy of the diet was evaluated using the PANDiet score, by comparing the nutrient intakes of 344 non-pregnant premenopausal women (18–44y) with dietary reference intakes for the first trimester of pregnancy. Simulations were performed to evaluate the theoretical efficiency of three types of ten successive tailored dietary changes in improving nutrient adequacy, with graded difficulty in implementation. The acceptability (declared intention to use in the diet) of most efficient dietary changes was evaluated during an online randomized study including 115 French pregnant women (22–41y).

**Results:**

Modifying the amount consumed of foods (type-1) did not modify the food repertoire and resulted in the smallest theoretical efficiency (increase in the PANDiet score of 9.8±0.2 points), but changes were the most acceptable (probability of the intention to use: 0.30–0.78). Conversely, replacing food items by items from the same group or eaten at the same time (type-3) broadened the food repertoire (3.6±1.3 food subgroups added) and resulted in the greatest theoretical efficiency (+23.9±0.3) but changes were the least acceptable (0.07–0.23). Replacing food items within the same subgroup (type-2) slightly broadened the food repertoire (+8.0±1.3 foods) and resulted in moderate theoretical efficiency (+14.8±0.2) and intermediate acceptability (0.11–0.35).

**Conclusion:**

A clear trade-off exists between the theoretical efficiency and acceptability of dietary changes, with a graded broadening of the food repertoire.

## Introduction

The nutrient adequacy of the diet, i.e. sufficient but not excessive intake of nutrients, is critical to individuals’ health [1–3]. However, for adults living in developed countries, “unhealthy” dietary patterns, associated with a high intake of saturated fatty acids (SFA) and a low intake of vitamins and minerals, persist [4–6]. In the general population, women of childbearing age who are likely to become pregnant are of growing interest in terms of public health issues related to nutrition [7]. Indeed, there is an important body of evidence that associates maternal nutrition during preconception and pregnancy with birth outcomes [8] and the health of offspring during childhood and adulthood [9,10]. However, in developed countries, although pregnancy has been described as being associated with a rise in nutrition awareness [11, 12] and a will to adopt healthier behaviors [13–15], nutrient requirements are not always satisfied [16, 17]. In France, few data are available on nutrient intakes in pregnant women, but studies of different samples have revealed a high n6:n3 fatty acid ratio [18], a moderate iodine deficiency [19] and inadequate intakes in dietary fiber, folate, vitamin D, SFA and sodium [20]. Furthermore, a previously published simulation study carried out by our team [21] showed that compliance with generic dietary guidelines, such as consuming recommended snacks, is only partially effective in maintaining the nutrient adequacy of the preconceptional diet of French women. This calls for major changes to the diet of women so that they will meet the higher nutrient requirements associated with pregnancy (e.g. thiamin, riboflavin, niacin, vitamin B6, folate, vitamin B12, vitamin C, vitamin D, iodine and zinc during the first trimester) [21]. We recently developed an algorithmic model that identifies the best nutritional substitution of a food or beverage that forms part of the observed diet of an individual with a food or beverage from the same food group or subgroup [22]. Given that adapting advice to individual habits can improve the perception of interventions [23], resorting to tailored dietary advice has proved more efficient than generic dietary advice [24, 25]. However, dietary advice identified as being theoretically efficient needs to be acceptable (e.g. it should appear realistic to implement it in the diet) to the targeted population if it is to be effective in practice [26, 27]. Although women in the periconceptional period are keener to adopt healthier eating behaviors [11–15], we could anticipate that the efficiency of dietary advice might be limited by its acceptability. Our objective was therefore to identify the dietary changes most efficient in improving nutrient adequacy during pregnancy, and to investigate a possible trade-off with acceptability.

We therefore ran a simulation and optimization study to evaluate the theoretical efficiency of three different types of dietary changes in improving the nutrient adequacy of the diet for the first trimester of pregnancy, followed by a study to evaluate the acceptability (declared intention to use in the diet) of these dietary changes to pregnant women. Finally, we combined the two studies in order to analyze the degree of acceptability of these changes that might limit an improvement in the nutrient adequacy of the diet during pregnancy.

## Materials and methods

**Fig 1:**
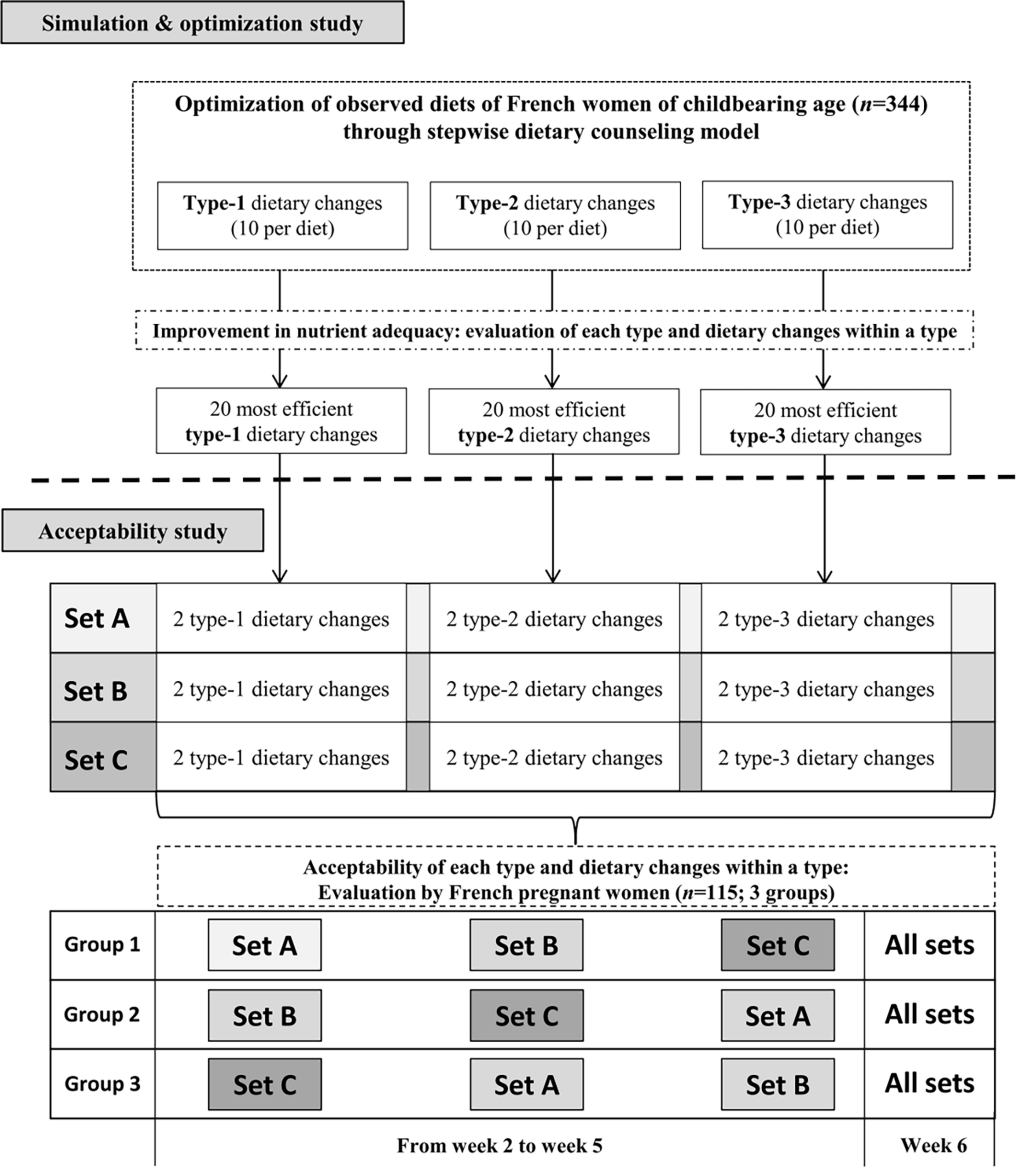
Combination between the simulation & optimization study and the acceptability study. Type-1 dietary changes consist in modifying the amount of a food item present in the observed diet (D0’). Type-2 dietary changes consist in replacing a food item consumed in the observed diet (D0’) by a food item from the same subgroup. Selection of food items to be used as potential substitutes under type-2 dietary changes was made to account for their consumption in the French population, their price and their seasonality. Type-3 dietary changes consist in replacing a food item consumed in the observed diet (D0’) by a food item from the same group or that could be consumed at the same time according to the French cultural meal scheme. For the simulation & optimization study (upper part), a step by step optimization process of each individual diet according to each type of changes was used, and this resulted in evaluating the efficiency of each type of changes and identifying the 20 most efficient changes of each type. In the acceptability study, each individual tested in total, 18 different dietary changes (6 of the most efficient of each type), that were divided into 3 sets composed by 6 dietary changes (two by type). Sets A, B and C were presented at three different occasions and each of the 18 changes was evaluated both a priori and a posteriori. Individuals were randomized in 3 groups (#1–3) for the order of evaluation of the 3 sets for the three occasions, according to a Latin square.

We report the results of two studies, a simulation & optimization study and an acceptability study, which are detailed below and the combination of their results are shown in **Fig 1**. Briefly, the simulation & optimization study allowed us to identify dietary changes that would be theoretically efficient in increasing the nutrient adequacy during the first trimester of pregnancy. The acceptability (as the declared intention to use in the diet) of these changes was then evaluated by a panel of French pregnant women during an online survey.

### Simulation & optimization study

#### Dietary data

Data were collected between February 2006 and July 2007 in the context of the French Nutrition and Health Survey (Etude Nationale Nutrition Santé, ENNS). The design and methods of the ENNS have been described in detail elsewhere [28]. The survey was carried out in compliance with the Declaration of Helsinki guidelines. All participants gave their written informed consent and the survey protocol was approved by an Ethics Committee (Hôpital Cochin, Paris, no. 2264) and the French Data Protection Authority (Commission Nationale de l’Informatique et des Libertés, CNIL, authorization no.905481). The authors of the present study were granted permission to use anonymized data from the ENNS survey by its principal investigator (Unité de Surveillance en Epidémiologie Nutritionnelle, USEN). The survey had a multistage, stratified, descriptive and cross-sectional design. Non-institutionalized 18 to 74-year-old people living in mainland France were selected at random and their dietary data were collected using three non-consecutive 24-hour recalls (including a weekend day) chosen randomly during a 2-week period. Dietary recalls were conducted over the telephone by trained dieticians. Nutritional values in terms of energy and nutrients were taken from a previously published nutrient database [29], updated as previously presented [21]. Mean individual nutrient intakes were calculated from food sources only, including a weighting for the day of the week (weekday or weekend day).

Subjects (i) who did not complete all three food recalls, (ii) who had missing information about variables required to calculate the nutrient adequacy index (namely sex, age, body weight, menstruation, pregnancy, lactation) and (iii) who were identified as under-reporters based on the method proposed by Black [30], were excluded. From the overall population, we selected women of childbearing age, defined as being premenopausal, not pregnant, not lactating and younger than 45 years of age (n = 344). The characteristics of these subjects are presented in **S1 Table**.

#### Nutrient adequacy assessment

The PANDiet aims to measure the overall diet quality of an individual by combining the probabilities of having an adequate nutrient intake. This intake is defined as the level likely to satisfy a nutrient requirement and unlikely to be excessive, according to dietary reference values, which differ according to age, sex and physiological condition [31]. The construction and design of the initial PANDiet and its updated version have been described in detail elsewhere [21, 32]. Briefly, the PANDiet is a 100-point score that results averaging two sub-scores, the Adequacy sub-score (Adeq-S) and the Moderation sub-score (Mod-S), so the higher the PANDiet score, the better the nutrient adequacy. The composition of each sub-score is detailed in **S1 Text**. The dietary reference values used to calculate the PANDiet were mostly those issued by the French Agency for Food, Environmental and Occupational Health (ANSES) (**S2 Table**). As previously described, for our study, the PANDiet score was adapted for early pregnancy in order to evaluate the nutrient adequacy of women of childbearing age during the first trimester of pregnancy. Therefore, the dietary reference values retained were those for pregnancy, except when they were identified as being specific to the third trimester [21, 33].

#### Stepwise dietary optimization model

We used a stepwise dietary optimization model designed to improve the initial simulated-pregnancy PANDiet score observed for each individual. The same process was implemented for the three types of dietary changes. Step 1 consisted in calculating the PANDiet score for pregnancy for the initial observed diet (D0). Step 2 involved the potential replacement of 134 food items not recommended during pregnancy and included in each observed diet with its cooked version for raw meat when available in the database (n = 4), with a very similar food item when identifiable (n = 3) or with an equivalent “average food” (n = 127), the latter being a theoretical food whose nutritional composition is the mean of that of similar food items weighted by their consumption in the population. The diet thus obtained was called the “initial observed modified diet” (D0’) and its associated PANDiet score was calculated. Step 3 then consisted in generating all possible dietary changes (depending on the selected type) with respect to D0’. Step 4 consisted in selecting the dietary change that enabled the greatest improvement in the PANDiet score while not decreasing energy intake versus D0’ or increasing it by more than 150 kcal. This was in line with the French guidelines which consider a 150-kcal increase in energy intake during the first trimester of pregnancy to be an approximate reference value [33]. Subsequent steps consisted in repeating the same actions (steps 3 and 4), starting from the diet that had been generated at the last step by retaining the dietary change enabling the greatest improvement.

During this study, we arbitrarily restricted the final number of dietary changes to 10 because pregnancy corresponds to a short period that limits the number of dietary changes possible and because our previous work in the general population had found that only minimal improvements in the PANDiet score were achieved beyond 10 steps [22].

#### Types of dietary changes

The selection of food items that were used for dietary changes was made from the ENNS food list (1427 foods and beverages divided into 14 groups and 66 subgroups) plus the 17 average food items that were created for step 2.

Briefly, three types of dietary changes were studied, with type-1 based on the modification of the amount consumed of a food item; and types 2 and 3 based on the replacement of a food item consumed in the observed diet by a food item either from the same subgroup (type-2) or from the same group or that could be consumed at the same time (type-3) (**Fig 1**). Details of the three types of dietary change and the numbers of food items by subgroup used for each type are presented in **S2 Text, S1 Fig and S3 Table** respectively.

Simulations and optimizations resulted in three pathways comprising 10 optimized dietary changes for each of the 344 women. Theoretical efficiency was defined as the mean improvement in the nutrient adequacy obtained for each dietary change or for a type of dietary change (after 10 changes).

### Acceptability study

#### Acceptability of dietary changes in pregnant women

We designed a 6-week online longitudinal survey to evaluate the acceptability of the dietary changes (and their associated types) identified by the simulation as being the most efficient. In total, 36 dietary changes (12 by type of dietary change) were selected to build three sets of dietary changes. In order to propose changes that targeted different food subgroups, the 36 dietary changes were selected from the 60 most frequently identified by the simulation (20 by type of dietary change). Dietary changes were assigned to one or another set (A, B or C) so that a participant would not evaluate two dietary changes in the same subgroup.

This study was approved by the Comité de Protection des Personnes Ile-de-France X (Study NI-2016-03-02), a French Ethics Committee. The women recruited for the study were members of an online panel operated by a generalist market research company (QualiQuanti), aged from 18 to 44 years and living in mainland France (n = 17,244). After eligibility was checked, women signed a consent form electronically. Non-inclusion criteria were as follows: not pregnant, more than six months pregnant, multiple pregnancy, specific diet linked to the dietary management of metabolic disorders or major food exclusions and no signature of the consent form. Eligible participants (n = 155) were asked to answer a questionnaire on their usual consumption of the 56 (out of 66) food subgroups involved in the dietary changes. This questionnaire was mainly used to avoid proposing the evaluation of a dietary change that might be irrelevant to their diet. If the change concerned a food item not usually consumed by the participant, a replacement dietary change was proposed. The final sample was composed of 115 women (full subject flow is shown in **S2 Fig**), who were randomly allocated to three groups using the survey and data analytics software Sphinx (Le Sphinx, Chavanod, France). Each group, of similar characteristics (**S4 Table**), evaluated the sets of dietary changes in a different order, and each participant evaluated independently the six dietary changes comprising the set. The endpoint to assess the acceptability of a dietary change was a declared intention to use it in the diet (“I might apply this dietary change to my diet once a week”: Yes–No). The evaluation was made both a priori (when the set was shown to the participant for the first time) and a posteriori (after a week-long period when she had time to consider more practically whether it may be difficult or not to implement the dietary change in her diet). Details about sequencing of evaluations for each group are provided in **Fig 1**. At the end of the study, the motivation of each participant was assessed in terms of the number of completed questionnaires. Participants who evaluated at least once one set of dietary changes (n = 106) were considered for the analysis (**S2 Fig**). Only 29 dietary changes (out of 36) which were evaluated at least five times a priori and five times a posteriori, were considered for the analysis.

### Statistical analysis

In the simulation study, descriptive statistics (mean, median, SD, SE and quartiles) were used to report PANDiet scores, associated sub-scores, probabilities of adequacy and changes relative to the three final simulated diets (D1, D2, D3) and the initial observed diets (D0 and D0’). These variables were not all normally distributed according to standard tests (such as the Kolmogorov-Smirnov test); however, according to the central limit theorem, and taking account of the number of subjects (*n* = 344), we used the normal approximation for performing mean comparison tests [34]. Student t-tests with a Bonferroni correction were performed to determine whether the means of the delta between the PANDiet scores, Adeq-S, Mod-S, probabilities of adequacy for nutrient intakes and energy intake excluding alcohol (EIEA) between D0 and the D0’, named Δ0’, and between D0’ and D1, D2 and D3, respectively named Δ1, Δ2 and Δ3, were different from 0. Sign tests with a Bonferroni correction were also performed to determine whether the percentage of individuals with a positive or negative Δ0’, Δ1, Δ2 and Δ3 for variables presented above, were different. Multiple mean comparisons were performed under a mixed model, taking a subject effect into account, with a Bonferroni correction to compare the means of Δ0’, Δ1, Δ2 and Δ3 for variables presented above.

In the acceptability study, descriptive statistics (mean, SD and range) were used to describe the declared intention to use dietary changes. A binomial logistic regression model with random effects for the subject was used to assess respectively whether the declared intention to use (2 levels) was associated with dietary changes (29 levels) or the type of dietary changes (1, 2 or 3), group (1, 2 or 3), order of evaluation (*i*.*e*. 1, 2 or 3), a priori/a posteriori (binary variable), number of completed questionnaires (from 1 to 6) and parity (binary variable: primiparous or multiparous). The probability that a participant might intend to use a dietary change was derived from the binomial logistic regression model. Correlations between evaluations a priori and a posteriori were evaluated using Pearson coefficients.

Combining the results of both studies, the relationship between the probability that a participant might intend to use a dietary change (“acceptability score”) and the mean increase in the PANDiet score obtained for the dietary change during the simulation study (“theoretical efficiency score”) was analyzed using linear regression with an inverse transformation of the theoretical efficiency score.

All analyses were performed using SAS 9.1.3 (version 9.1.3, SAS Institute Inc., Cary, NC). *P*<0.05 were considered to be statistically significant.

## Results

### Analysis of the preliminary step before simulations: Replacement of the food items not recommended during pregnancy

Briefly, this step barely modified the quality of the diet: from D0 to D0’, there was only an increase of 1.15% in the PANDiet score (P<0.001). Details are shown in **S5 Table**.

### Analysis of the kinetics of PANDiet scores, associated sub-scores and energy intake variations between the initial observed modified (D0’) and final simulated (D1, D2, D3) diets

**Fig 2:**
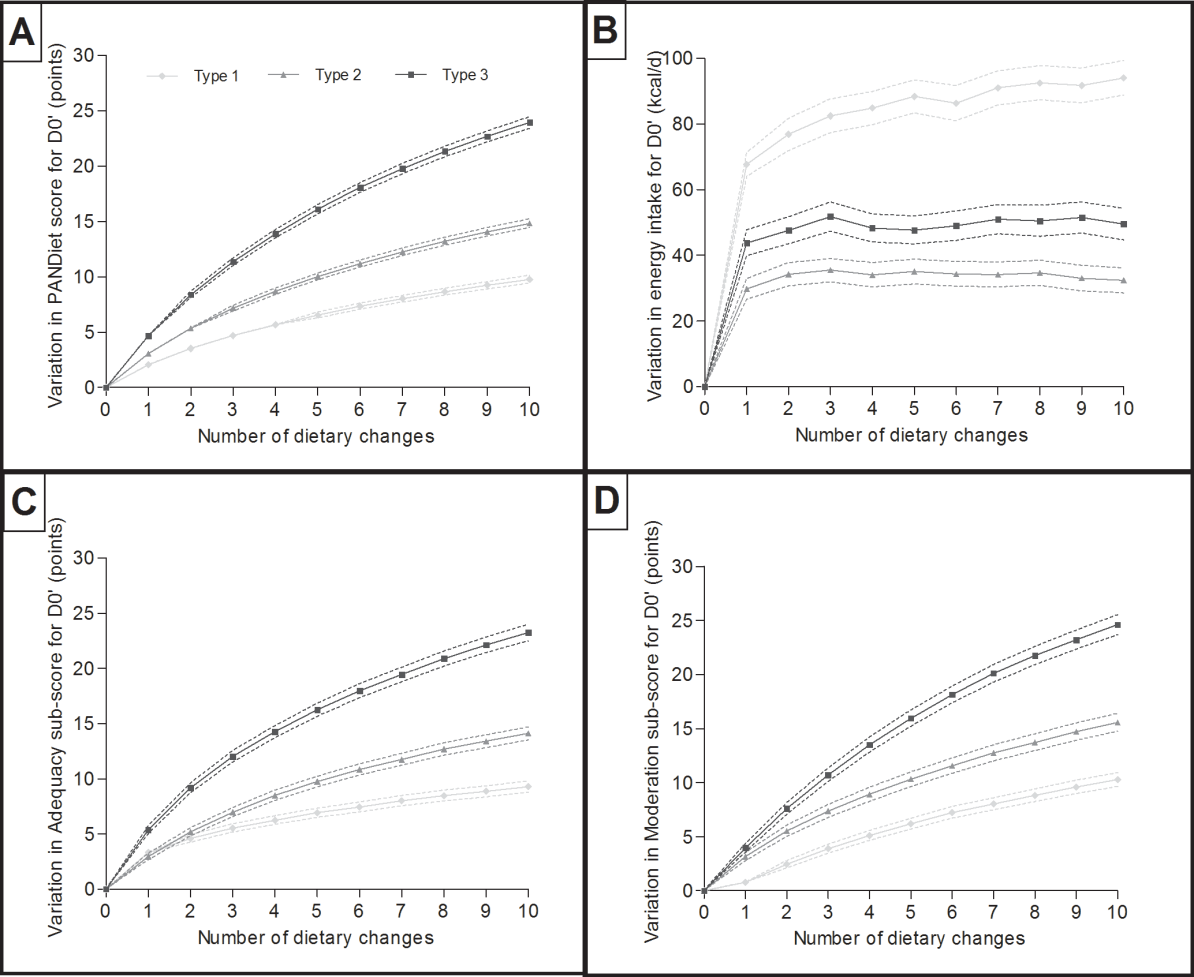
**Changes to the PANDiet score (Panel A), energy intake excluding alcohol (EIEA, Panel B), Adequacy sub-score (Adeq-S; Panel C) and Moderation sub-score (Mod-S; Panel D) between the initial observed modified diet (D0’) and the three final simulated diets (D1, D2, D3) in line with the ten subsequent dietary changes.** Solid lines represent the variation in the mean and dashed lines represent 95% CIs. n = 344.

At the end of the ten steps of the stepwise dietary counseling model, three final simulated diets (D1, D2 and D3) were obtained for each of the 344 women, corresponding to the three different types. The changes in the PANDiet scores, Adeq-S, Mod-S and energy intakes excluding alcohol (EIEA) of the diets relative to all ten dietary changes are presented in **Fig 2**. After ten dietary changes, the increases in the PANDiet score were, in descending order, 23.9±0.26 with type-3 dietary changes, 14.8±0.20 with type-2 dietary changes and 9.78±0.18 with type-1 dietary changes (*Ps*<0.001). So, theoretical efficiency rose from type-1 to type-3. Increases in Adeq-S and Mod-S ranked the same between the three different types of dietary changes. Increases in EIEA were, in descending order, 94.0±2.65 with type-1 dietary changes, 49.5±2.42 with type-3 dietary changes and 32.3±1.94 with type-2 dietary changes (*Ps*<0.001). There was an effect of the PANDiet quartile of D0’ on Δ1 (*P*<0.01), Δ2 (*P*<0.05) and Δ3 (*P*<0.001), respectively. The lower the PANDiet of D0’, the higher were the Δ1, Δ2 and Δ3 values. More details can be found in **S6, S7, S8 and S9 Tables**.

### Analysis of the nutrient adequacy of the initial observed modified (D0’) and final simulated (D1, D2, D3) diets

**Fig 3:**
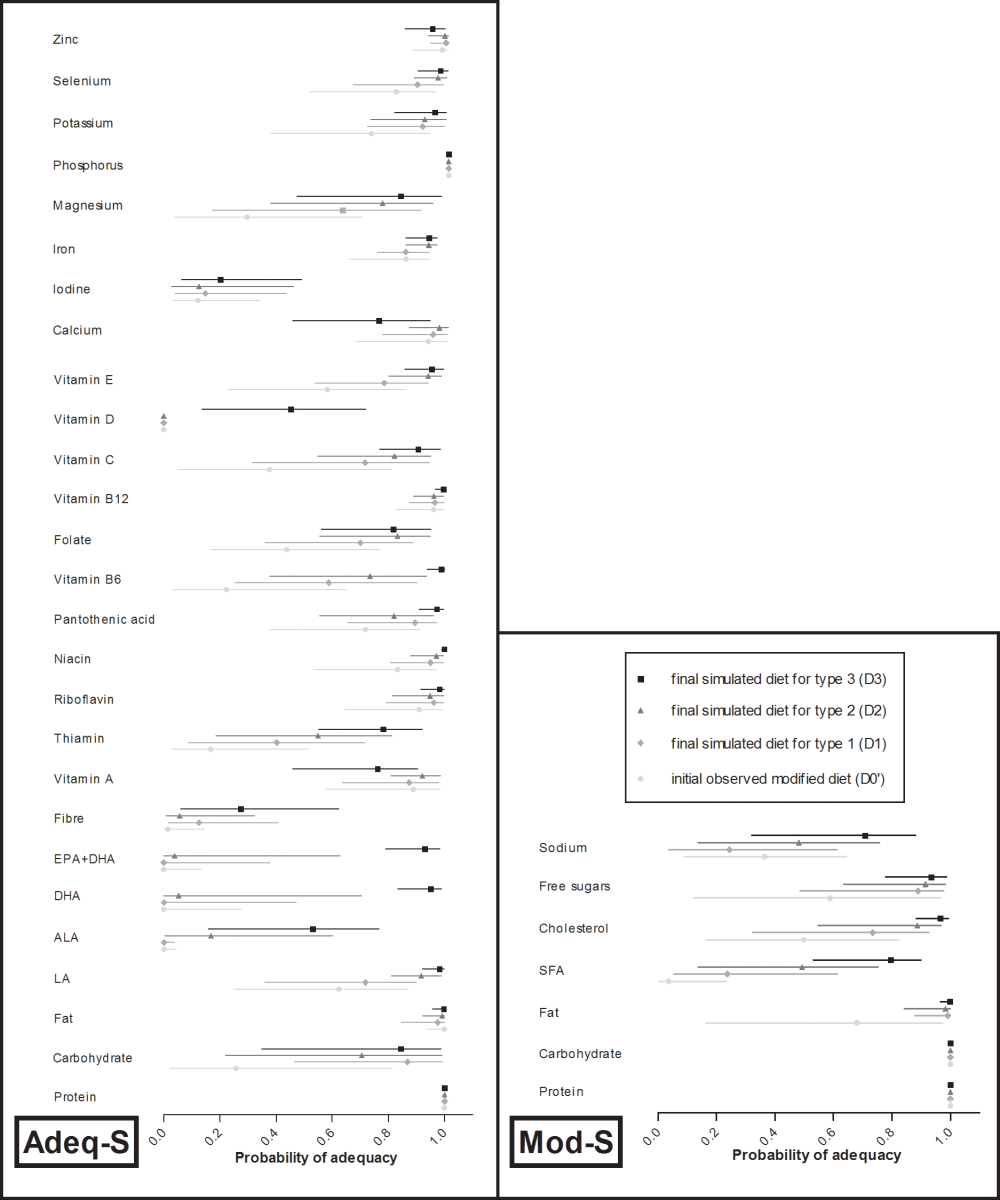
Distribution (first quartile, median, and third quartile) of the probability of adequacy for each nutrient intake considered in the adequacy sub-score (Adeq-S) and in the Moderation sub-score (Mod-S) for the observed initial modified diets (D0’) and the final simulated diets with the three types of dietary changes (D1, D2, D3) separately (Etude Nationale Nutrition Santé 2006–2007). The starting point of each line represents the quartile 1 (25^th^ percentile), the mark (filled circle for D0, filled diamond for D1, filled triangle for D2 and filled square for D3) on each line represents the median, and the final point of each line represents the quartile 3 (75^th^ percentile). The light gray lines with light gray circles represent D0’, the medium gray lines with medium gray diamonds represent D1, the dark gray lines with dark gray triangles represent D2 and the black lines with black squares represents D3. n = 344.

An increase in the probability of adequacy was observed for 28, 30 and 31 nutrient intakes with types -1, -2 and -3 dietary changes, respectively (**Fig 3**). Globally, the variation in the probabilities of adequacy for nutrient intakes increased significantly from type-1 dietary changes to type-2 dietary changes and then type-3 (total fat, LA, ALA, DHA, EPA+DHA, thiamin, vitamins B6, C, D and E, iron and magnesium intakes in the Adeq-S (12 out of 27) and SFA, cholesterol and sodium intakes (3 out of 7) in the Mod-S; see **S6, S7, S8 and S9 Tables**). For dietary fiber, pantothenic acid and potassium, the variation in the probabilities of adequacy was higher with type-1 dietary changes than with type-2. An increase in the probability of adequacy regarding vitamin A and calcium intakes was observed with dietary changes of type 1 (except for vitamin A) and of type 2, whereas a decrease was observed with type-3 dietary changes. Very small mean increases in the probability of adequacy for iodine were found, but approximately half of the women increased their adequacy with type-2 dietary changes, and approximately two thirds with types-1 and -3 dietary changes.

Under type-1 dietary changes, foods belonging to the “Bread”, “Fruits, fresh”, and “Vegetables, raw” subgroups were the main contributors to dietary changes with increases in the amounts consumed, whereas foods from the “Butter, margarine and fresh cream” and “Ripened cheese” subgroups were the main contributors to those with decreases. Under type-2 dietary changes, food belonging to the “Butter, margarine and fresh cream”, “Bread”, “Meat, unprocessed” and “Vegetables, cooked” subgroups were the main contributors to substitutions. A mean of 8.0 ± 1.3 foods which were not initially consumed by the women were added to their food repertoire. Under type-3 dietary changes, food items from the “Fish, unprocessed”, “Breakfast cereals”, “Nuts, oilseeds and oil fruits”, and “Fruits, fresh” subgroups were the most frequently used as substitutes, whereas foods from the “Ripened cheese”, “Bread” and “Meat, unprocessed” subgroups were the most frequently substituted. A mean of 3.6 ± 1.3 food subgroups were added to their food repertoire.

### Analysis of the acceptability of dietary changes and types of dietary changes

The declared intention to use a priori and a posteriori were well correlated (Pearson correlations, ρ = 0.60, *P*<0.001). The 29 dietary changes were ranked according to the probability of their declared intention to use, derived from the binomial logistic regression model. The 11 type-1 dietary changes were ranked from first to twelfth (1–9, 11 and 12) with a mean probability of declared intention to use of 0.51±0.14 (range: 0.30–0.77). The eight type-2 dietary changes were ranked from tenth to twenty-sixth (10, 13, 14, 19, 21, 22, 24 and 26) with a mean probability of declared intention to use of 0.22±0.08 (range: 0.10–0.35). The ten type-3 dietary changes were ranked from fifteenth to twenty-ninth (15, 16–18, 20, 23, 25, 27–29) with a mean probability of declared intention to use of 0.1±0.06 (range: 0.07–0.23). Among the other variables included in the model (group, order of evaluation, a priori/a posteriori, number of questionnaires and parity), only parity had a significant effect on declared intention to use. When introducing potential confounding factors other than parity into the model (age, number of people composing the household, number of children, socio-professional category, occupation, urbanization of the place of residence), there were no changes greater than one rank.

### Comparison of efficiency in improving the nutrient adequacy of the diet and the acceptability of dietary changes

**Fig 4:**
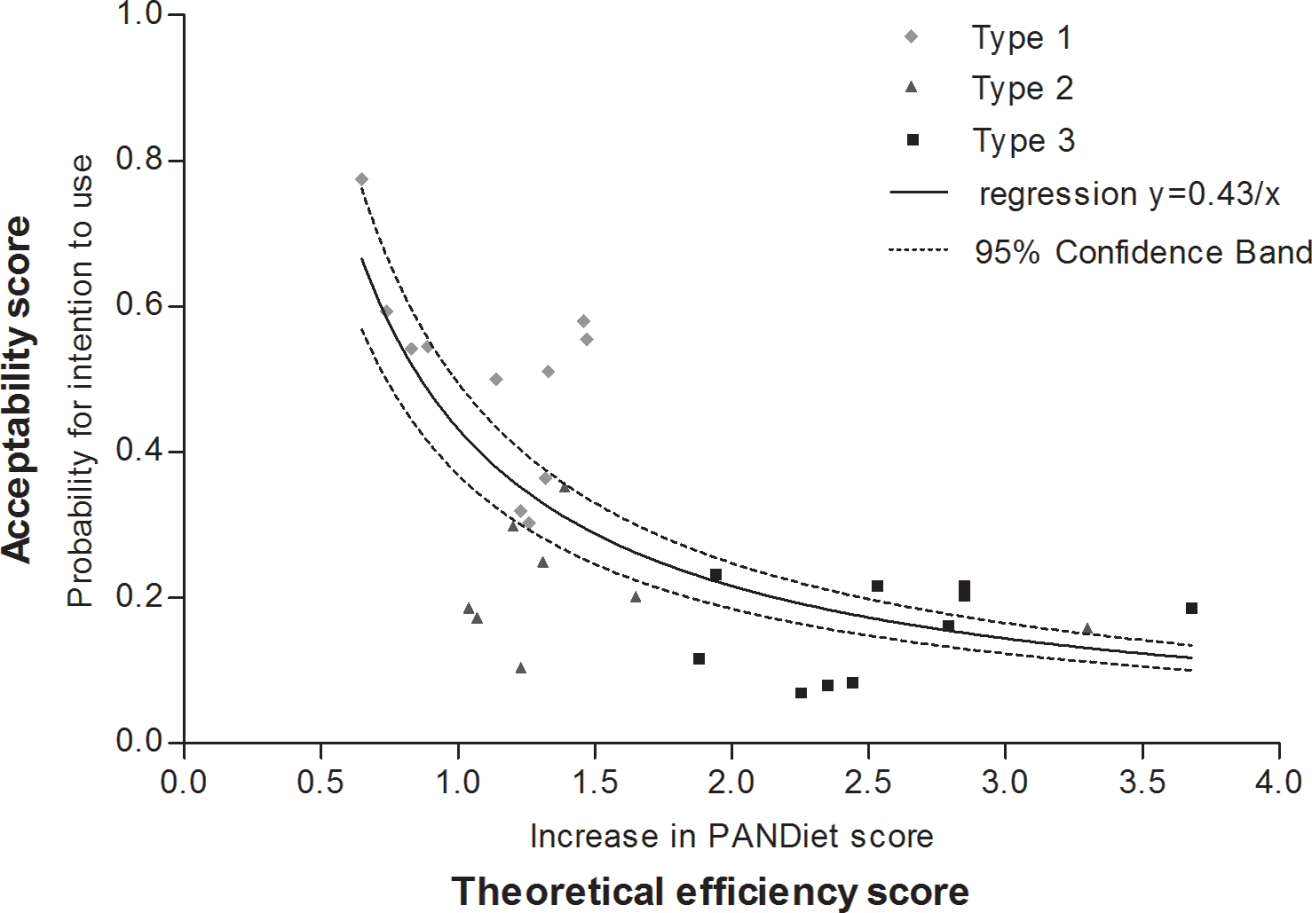
Acceptability of dietary changes (y axis) according to their theoretical efficiency (x axis). Acceptability was evaluated as the probability of the declared intention to use derived from the binomial logistic regression model (i.e. the probability that participants intended to use a dietary change) for the acceptability study (n = 106). Theoretical efficiency was evaluated as the mean increase in the PANDiet score (points) obtained with this dietary change in the simulation study (n = 344, Etude Nationale Nutrition Santé, 2006–2007). Evaluations of acceptability and theoretical efficiency are presented for 29 dietary changes. Mid-gray diamonds represent type-1 dietary changes (n = 11), dark gray triangles represent type-2 dietary changes (*n* = 8) and black gray squares represent type-3 dietary changes *(*n = 10). The solid line represents the regression curve with the following equation: y = 0.43/x, R^2^ = 0.54, and the dashed lines represent the 95% CIs for this curve.

**Fig 4** presents the relationship between the “acceptability score” of dietary changes (evaluated as the probability of declared intention to use) and their “theoretical efficiency score” (evaluated as the mean increase in the PANDiet score observed with this dietary change during simulations). The relationship between acceptability and theoretical efficiency was not linear and fitted well with the simple one-parameter inverse function (Acceptability = 0.43/Theoretical efficiency, R^2^ = 0.54; **Fig 4**). Type-1 dietary changes were the least efficient but the most acceptable, while conversely, type-3 dietary changes were the most efficient but the least acceptable. The ranges of theoretical efficiency for type-1 and type-2 dietary changes were 18–40% and 28–90%, respectively, of the most efficient type-3 dietary change. The ranges of acceptability of type-2 and type-3 dietary changes were 13–45% and 9–30%, respectively, of the most acceptable type-1 dietary change. Type-2 dietary changes had an intermediary range of rating for efficiency and acceptability, and ultimately a lower overall rating when acceptability and efficiency were considered together (as illustrated by their central position in the concavity of the regression curve, **Fig 4**). Overall, the more dietary changes were efficient in improving the nutrient adequacy of the diet, the less they were acceptable, but the relation was variable and not linear.

## Discussion

During this study, we compared different types of dietary changes to improve nutrient adequacy during pregnancy using a tailored approach, and were able to analyze the relationship between the theoretical efficiency and acceptability of these changes. The three types of tailored dietary changes had different a priori graded difficulty in implementation by individuals ranging from type-1 where no new foods were added to the initial diet, to type-2 where a new food item could be added to the food repertoire providing it belonged to a food subgroup that was already being consumed by the individual, and type-3 where a food item belonging to new food subgroups could be added to the food repertoire (providing that it belonged to the same group or could be consumed at the same time). In type-1 dietary changes, a broadening of the food repertoire remained impossible and the individual's eating habits were preserved. By contrast, type-2 dietary changes produced diets containing a mean of eight supplemental food items (out of a possible ten) and no new food subgroups (by construction) and type-3 dietary changes generated diets containing a mean of four supplemental food subgroups (out of a possible ten). The type-2 dietary changes were substitutions limited to a particular food subgroup, thus their impact on the individual's eating habits remained small. By contrast, type-3 dietary changes were based on substitutions between food items from the same group or consumed at the same time, enabling a greater broadening of the food repertoire. As expected, the types of dietary changes which enabled more broadening of the food repertoire were the most efficient in improving the nutrient adequacy of the diet. In the online survey, the type which least broadened the food repertoire was found more acceptable by pregnant women. However, the relationship between efficiency and acceptability was not linear. Our analysis of this trade-off was able to show that in general, the final expected impact of a higher theoretically efficient change is largely limited by the lower acceptability.

Furthermore, the tailored approach used here appeared to be more efficient than a generic approach. In particular, tailored dietary changes based on the observed diet resulted in a greater increase in the simulated-pregnancy PANDiet score after four (type-1) and two (types -2 and -3) dietary changes than adding any of the 150-kcal snacks recommended during pregnancy and also simulated in the same population, as reported elsewhere [21].

It is important to note that even though the type-1 dietary changes did not broaden the food repertoire and resulted in only a minor improvement in overall nutrient adequacy as compared to the other types of dietary changes, they could still improve the overall nutrient adequacy of women at the start of their pregnancy, by balancing the amounts of different foods consumed. They could even be more efficient than type-2 dietary changes in improving the intake of certain nutrients. For instance, the probability of an adequate dietary fiber intake, which is low in France and other western countries [35, 36], was more increased with type-1 than with type-2 dietary changes.

The type-2 dietary changes were surprisingly efficient in improving overall nutrient adequacy at the beginning of pregnancy, inasmuch as we limited the extent of substitutions (based on the level of consumption of food items in the French population, their price and seasonality). Overall, the type-2 dietary changes improved the probability of adequacy for all nutrient intakes in most of the population, except for some specific nutrients (such as vitamin D and iodine, whose intakes were markedly inadequate). When focusing on folate, whose requirement fulfillment at the start of pregnancy is very important [37], the type-2 dietary changes were more efficient than type-1 changes in improving the probability of adequate intake.

The marked efficiency of type-3 dietary changes could be ascribed to a greater use of food items belonging to the “Fish, unprocessed” and “Nuts, oilseeds and oil fruits” subgroups, the consumption of which remains low in the French population [38]. Introducing foods from these subgroups contributed to dramatically improving the intake of long chain PUFA and vitamin D. This could be seen as a major strength of this type of dietary change, knowing that an adequate intake of omega-3 fatty acids can impact pregnancy outcomes [39], human milk composition and the DHA status of offspring [40]. However, with type-3 dietary changes, the slight decrease in the probabilities of adequacy for calcium and vitamin A intakes could be ascribed to substituting food items from the “Dairy” group with those from the “Fruits and nuts” group, and from the “Butter, margarine and fresh cream” subgroup with those from the “Oil” subgroup. Such slight reductions could be considered as side effects of the global approach used in our dietary counseling model, which was driven by increasing the probability of overall nutrient adequacy of the diet, not the intake of a specific nutrient.

A comparison of the efficiency results achieved with each type of dietary change revealed that broadening the usual food repertoire was necessary to meet nutrient requirements, a finding which had already been highlighted during a diet modeling study in French adults [41]. Here we could show that broadening the food repertoire was deemed less acceptable. This lower acceptability should be expected to be a firm limit to the efficiency of that type of dietary change for improving nutrient adequacy of the diet.

### Strengths and limitations

There are no data comparing the nutritional efficiency of tailored dietary changes with a priori graded difficulty of implementation and studying their acceptability in pregnant women. In this group, dietary interventions have mostly targeted those who are overweight and/or obese and/or at risk of gestational diabetes. In most cases, dietary counseling has been based on generic dietary guidelines (e.g. increasing the consumption of fruits and vegetables, increasing dietary fiber intakes, and reducing those of SFA and refined carbohydrates); to some extent they have been adapted for pregnant women [42, 43], but not tailored to the adequacy of their nutrient intakes in view of the specific nutrient requirements for pregnancy. By contrast, our study was conducted in a general population of women, and identified dietary advice based on theoretical efficiency (using a tailored approach) and acceptability (using a dedicated online survey). A major strength of our approach was the dual assessment of efficiency and acceptability, considering the trade-offs that could exist between them as a function of the type of changes.

The principal limitation of this study was its theoretical aspect because we used a dietary optimization model to generate theoretical best changes for being tested in the acceptability study. Although three days of record is a standard, relevant method in dietary surveys, and has proven adequate to score the diet of the individuals with the PANDiet system, it is not able to capture food items that are less frequently consumed and this could have slightly limited the number of dietary changes that could have been studied. Then, it can be noted that the food grouping used in types-2 and -3 dietary changes had an impact on the dietary changes that were the most proposed by our algorithm, but it corresponds to the classification used by ANSES in France. Furthermore, we used dietary data from women of childbearing age, whereas we could hypothesize that if they had been pregnant, some of the women would have adopted a healthier diet [11]. Nonetheless, the approach remains relevant to women of childbearing age who are considering changes to their diet during the periconceptional period, because we were able to show that at the start of pregnancy, just a few tailored dietary changes to the pre-pregnancy diet would result in marked improvements in nutrient adequacy. As some confounders that could impact dietary intakes such as medical conditions, physical activity or gestational weight gain were not collected in the acceptability study because women were not followed all along their pregnancy, they could not be included in the statistical analysis, and this could be a limitation of this part of the study. Then, evaluating the acceptability of a dietary change as the intention to use it could be considered as a limitation since it is a general endpoint. In the future, more specific dimensions of the acceptability could be evaluated (such as the cost, the cooking skills and the preferences). The motivation of women to modify their dietary habits during pregnancy could also be evaluated using a dedicated behavior change theory. Lastly, because we needed first of all to design different types of dietary changes and evaluate their efficiency in improving the nutrient adequacy of the diet, efficiency and acceptability could not have been evaluated in the same samples.

## Conclusion

In conclusion, we characterize a clear trade-off between theoretical efficiency and acceptability of dietary changes with a graded broadening of the food repertoire, in order to improve nutrient adequacy during pregnancy in French women: the type of dietary changes that most broadened the food repertoire and most improved the nutrient adequacy was also the least acceptable. This trade-off has practical implication for the design of dietary interventions that would be practically effective in improving the nutrient adequacy of pregnant women.

## Supporting information

S1 Table Characteristics of women of childbearing age (premenopausal, 18–44y, *n* = 344) from the ENNS^1^ survey. (DOCX)

S2 Table Items, reference values^1^ and variabilities used to calculate the PANDiet score at the start of pregnancy. (DOCX)

S3 Table Number of food items by subgroups in total, and number of food items whose the amount consumed could be modified with type-1 dietary changes, whose the substitution is possible with types -2 and -3 dietary changes, and that could be used as a substitute in types -2 and -3 dietary changes. (DOCX)

S4 Table Characteristics of pregnant women (*n* = 115) included in the acceptability study of dietary changes during pregnancy. (DOCX)

S5 Table PANDiet scores, Adeq-S, Mod-S, probabilities of adequacy for nutrient intakes and total energy intake excluding alcohol for the initial observed diet (D0), the initial observed modified diet (D0’), their delta (Δ0’) and the percentage of individuals with an increase between D0’ and D0 for women of childbearing age (*n* = 344) participating in the ENNS^1^ study. (DOCX)

S6 Table PANDiet score, Adeq-S, Mod-S, total energy intake without alcohol and probabilities of adequacy for nutrient intakes for D0’ (initial observed modified diet) and their changes in final simulated diets under type-1 (Δ1), type-2 (Δ2) and type-3 (Δ3) changes for women of childbearing age (*n* = 344) participating in the ENNS^1^ study. (DOCX)

S7 Table PANDiet scores, Adeq-S, Mod-S, probabilities of adequacy for nutrient intakes and total energy intake excluding alcohol for the initial observed modified diet (D0’), the final simulated diet under type-1 dietary changes (D1), their delta (Δ1) and the percentage of individuals with an increase between D1 and D0’ for women of childbearing age (*n* = 344) participating in the ENNS^1^ study. (DOCX)

S8 Table PANDiet scores, Adeq-S, Mod-S, probabilities of adequacy for nutrient intakes and total energy intake excluding alcohol for the initial observed modified diet (D0’), the final simulated diet under type-2 dietary changes (D2), their delta (Δ2) and the percentage of individuals with an increase between D2 and D0’ for women of childbearing age (*n* = 344) participating in the ENNS^1^ study. (DOCX)

S9 Table PANDiet scores, Adeq-S, Mod-S, probabilities of adequacy for nutrient intakes and total energy intake excluding alcohol for the initial observed modified diet (D0’), the final simulated diet under type-3 dietary changes (D3), their delta (Δ3) and the percentage of individuals with an increase between D3 and D0’ for women of childbearing age (n = 344) participating in the ENNS^1^ study. (DOCX)

S10 Table Application of the STROBE-nut (An extension of the STROBE statement for nutritional epidemiology, developed by Lachat C *et al*. (2016)) to our studies. (DOCX)

S11 Table Acceptability study–minimal data set. (XLSX)

S1 Text Details of each sub-score composing the PANDiet score. (DOCX)

S2 Text Details about the three types of dietary changes used in the simulation study. (DOCX)

S1 Fig Diagram presenting possible substitutions depending on the meal between food subgroups belonging to different food groups under type-3 dietary changes. The name of the food group is presented in bold in the first row of each box. The names of the food subgroups belonging to the group are presented from the second row to the last row of the box. Food items belonging to food subgroups whose name is written in italics and underlined could be used as substitutes, but only for lunch and dinner. Food items belonging to food subgroups whose name is written in italics, underlined and followed by an asterisk (*) could be used as substitutes, but only for lunch, dinner and snack. Food items belonging to food subgroups whose name is written in italics could be used as substitutes, but only for breakfast and snack. Food items belonging to food subgroups whose name is written without specific typesetting could be used as substitutes, for all meals. Dietary changes between food subgroups belonging to boxes linked by dotted-dashed arrows could only be made for lunch and dinner. Dietary changes between food subgroups belonging to boxes linked by dashed arrows could only be made for breakfast and snack. Dietary changes between food subgroups belonging to boxes linked by continuous arrows could be made for all meals.
(DOCX)

S2 Fig Diagram of subject flow and reasons for non-inclusions in the study of the acceptability of dietary changes during pregnancy. (DOCX)
