# Supplementary material for: A clear trade-off exists between the theoretical efficiency and acceptability of dietary changes that improve nutrient adequacy during early pregnancy in French women: Combined data from simulated changes modeling and online assessment survey
Source: PLoS One. 2018 Apr 11;13(4):e0194764. doi: 10.1371/journal.pone.0194764 (PMC5895017; doi:10.1371/journal.pone.0194764)
Supplement: S1 Table — (DOCX) [file pone.0194764.s001.docx]

**S1 Table.** Characteristics of women of childbearing age (premenopausal, 18-44y, *n*=344) from the ENNS^1^ survey

|  | Total  (*n*=344) |
| --- | --- |
| Age^2^ (years) | 35.1 ± 6.9 |
| Number of people composing the household^3^  1  2  3  4  5  6 or more | 9.0% (31)  16.3% (56)  18.6% (64)  38.4% (132)  12.8% (44)  4.9% (17) |
| Number of children^3^  0  1  2  3  4 or more  Absence of answer | 24.1% (83)  16.6% (57)  38.1% (131)  15.7% (54)  5.2% (18)  0.3% (1) |
| Occupation^3^  Farmer, Craftsperson, Storekeeper  Professional, executive  Intermediate profession  Employee  Manual worker  Student  Unemployed | 3.8% (13)  17.4 % (60)  27.6% (95)  36.3% (125)  8.7% (30)  4.9% (17)  1.2% (4) |
| Socio-professional category^3,4^  High  Low  Inactive | 48.8% (168)  45.1% (155)  6.1% (21) |
| Urbanization of the place of residence^3^  Paris  Major city (>100,000 inhab.)  Medium-sized town (20 – 100,000 inhab.)  Small-sized town (2 – 20,000 inhab.)  Rural area | 15.4% (53)  31.4% (108)  15.1% (52)  15.7% (54)  22.4% (77) |

^1^ *Etude Nationale Nutrition Santé*, 2006-2007.

^2^ Values are mean ± SD

^3^ Values correspond to the percentage of participants presenting the characteristic described in the first column followed by the associated number of participants in parentheses.

^4^ Socio-professional categories were derived from occupations. “Farmer, craftsperson, storekeeper”, “Professional, executive”, and “Intermediate profession” belong to the High socio-professional category, “Employee”, “Manual worker” and “Student” belong to the Low socio-professional category and “Unemployed” belong to the Inactive socio-professional category.
