# Supplementary material for: A clear trade-off exists between the theoretical efficiency and acceptability of dietary changes that improve nutrient adequacy during early pregnancy in French women: Combined data from simulated changes modeling and online assessment survey
Source: PLoS One. 2018 Apr 11;13(4):e0194764. doi: 10.1371/journal.pone.0194764 (PMC5895017; doi:10.1371/journal.pone.0194764)
Supplement: S2 Table — (DOCX) [file pone.0194764.s002.docx]

**S2 Table.** Items, reference values^1^ and variabilities used to calculate the PANDiet score at the start of pregnancy.

|  | Pregnant women (1^st^  trimester) | Variability |
| --- | --- | --- |
| Adequacy sub-score | | |
| Protein | 0.66 g/kg bw | 12.50 % |
| Total Fat | 30% EIEA | 0 % |
| LA (C18:2 n-6) | 3.08% EIEA | 15 % |
| ALA (C18:3 n-3) | 0.77% EIEA | 15 % |
| DHA | 192 mg | 15 % |
| EPA + DHA | 385 mg | 15 % |
| Total carbohydrate | 45 % EIEA | 0 % |
| Dietary fiber | 25 g | 15 % |
| Vitamin A | 462 µg RE | 15% |
| Thiamin | 1.39 mg | 15% |
| Riboflavin | 1.23 mg | 15 % |
| Niacin | 12.3 mg NES | 15 % |
| Pantothenic acid | 3.85 mg | 15 % |
| Vitamin B6 | 1.67 mg | 10 % |
| Folate | 286 µg | 20 % |
| Vitamin B12 | 2.17 µg | 10 % |
| Vitamin C | 92.3 mg | 15 % |
| Vitamin D | 7.70 µg | 15 % |
| Vitamin E | 9.23 mg | 15 % |
| Calcium | 690 mg | 15 % |
| Iodine | 143 µg | 20 % |
| Iron | Based on tables provided by the IoM | |
| Magnesium | 5 mg/kg bw | 10 % |
| Phosphorus | 580 mg | 15 % |
| Potassium | 2385 mg | 15 % |
| Selenium | 38.5 µg | 15 % |
| Zinc | 6.41 mg | 15 % |
| Moderation sub-score | | |
| Protein | 2.2 g/kg bw | 0 % |
| Total carbohydrate | 60% EIEA | 0 % |
| Free sugars | 10% EIEA | 15 % |
| Total fat | 40% EIEA | 0 % |
| Saturated Fatty Acids | 12% EIEA | 15 % |
| Cholesterol | 300 mg | 15 % |
| Sodium | 2365 mg | 15 % |
| *Tolerable Upper Intakes Limits for potential penalties* | | |
| Retinol | 3000 µg | |
| Niacin | 900 mg | |
| Vitamin B6 | 25 mg | |
| Folate | 1000 µg | |
| Vitamin C | 500 mg | |
| Vitamin D | 50 µg | |
| Vitamin E | 300 mg | |
| Calcium | 2500 mg | |
| Iodine | 600 µg | |
| Iron | 28 mg | |
| Magnesium | 700 mg | |
| Phosphorus | 2500 mg | |
| Selenium | 300 µg | |
| Zinc | 25 mg | |

^1^References values were mostly issued by the French Agency for Food, Environmental and Occupational Health.

The Adequacy sub-score comprises 27 items and the Moderation sub-score comprises 7 items plus 14 potential penalty values.

ALA, Alpha Linolenic Acid. Bw, bodyweight. DHA, docosahexaenoic acid. EPA, eicosapentaenoic acid. EIEA, Energy Intake Excluding Alcohol. LA, Linoleic Acid. NES, Niacin Equivalents. RE, Retinol Equivalents
