# Supplementary material for: A clear trade-off exists between the theoretical efficiency and acceptability of dietary changes that improve nutrient adequacy during early pregnancy in French women: Combined data from simulated changes modeling and online assessment survey
Source: PLoS One. 2018 Apr 11;13(4):e0194764. doi: 10.1371/journal.pone.0194764 (PMC5895017; doi:10.1371/journal.pone.0194764)
Supplement: S3 Table — (DOCX) [file pone.0194764.s003.docx]

**S3 Table.** Number of food items by subgroups in total, and number of food items whose the amount consumed could be modified with type-1 dietary changes, whose the substitution is possible with types -2 and -3 dietary changes, and that could be used as a substitute in types -2 and -3 dietary changes.

|  |  | Number of food items | | | | | |
| --- | --- | --- | --- | --- | --- | --- | --- |
|  |  | Total^1^ | Type 1^2^ | Type 2^2^ | | Type 3^2^ | |
| Group | Subgroup |  | Amount consumed modifiable | Substitution possible | Potential substitute | Substitution possible | Potential substitute^3^ |
| Alcoholic beverages | | 36 | 0 | 0 | 0 | 0 | 0 |
| Biscuits, cakes and pastries | Salty snacks and crackers | 11 | 11 | 11 | 7 | 11 | 3 |
|  | Sweet biscuits and cereal bars | 38 | 37 | 38 | 16 | 38 | 5 |
|  | Cakes and grain-based desserts | 57 (1) | 49 | 49 | 21 | 49 | 7 |
|  | Ice cream and frozen desserts | 21 (1) | 19 | 19 | 8 | 19 | 7 |
|  | Viennoiseries and buns | 14 | 14 | 14 | 7 | 14 | 4 |
| Grains and starchy foods | Crisp breads and dried bread products | 19 | 16 | 17 | 7 | 17 | 1 |
|  | Breakfast cereals | 18 | 18 | 18 | 10 | 18 | 2 |
|  | Flours | 4 | 0 | 0 | 0 | 0 | 0 |
|  | Grain or starch-based main dishes | 34 | 27 | 34 | 12 | 34 | 5 |
|  | Bread | 13 | 12 | 13 | 6 | 13 | 4 |
|  | Pasta, rice and other cereals | 18 | 17 | 17 | 8 | 17 | 6 |
|  | Potatoes and similar products | 16 | 16 | 16 | 7 | 16 | 3 |
| Confectionery and chocolates | Cocoa, chocolate and chocolate products | 14 | 12 | 12 | 6 | 12 | 6 |
|  | Confectionery excluding chocolate | 17 | 15 | 15 | 8 | 15 | 3 |
| Jams, honey and other sugar products | Jam, honey and sweetened spreads | 7 | 7 | 7 | 4 | 7 | 4 |
|  | Sweetened sauces | 7 | 7 | 7 | 6 | 7 | 2 |
|  | Sugar | 1 | 1 | 0 | 0 | 0 | 0 |
| Water and beverages (non-alcoholic) | Sugar-sweetened beverages | 20 (1) | 15 | 15 | 7 | 15 | 2 |
|  | Coffee, tea and other hot beverages | 20 | 0 | 0 | 0 | 0 | 0 |
|  | Water | 32 | 0 | 0 | 0 | 0 | 0 |
|  | Fruit juices | 25 | 25 | 25 | 11 | 25 | 5 |
|  | Plant-based drinks (e.g. rice milk) | 5 | 5 | 5 | 3 | 5 | 1 |
| Fruits and seeds | Fruit in syrup and compotes | 15 | 15 | 15 | 6 | 15 | 3 |
|  | Fruits, fresh | 45 | 45 | 45 | 10 | 45 | 6 |
|  | Dried fruits and chestnuts | 11 | 11 | 11 | 9 | 11 | 1 |
|  | Nuts, oilseeds and oil fruits | 16 | 10 | 10 | 7 | 10 | 1 |
| Vegetables | Vegetable-based starters | 12 | 12 | 12 | 7 | 12 | 2 |
|  | Vegetables, raw | 31 | 30 | 31 | 14 | 31 | 9 |
|  | Vegetable-based main dishes | 17 | 17 | 17 | 9 | 17 | 2 |
|  | Vegetables, cooked | 40 | 39 | 39 | 18 | 39 | 9 |
|  | Legumes | 12 | 10 | 12 | 4 | 12 | 1 |
|  | Vegetable-based soups | 22 | 21 | 21 | 10 | 21 | 2 |
| Fats | Butter, margarine and fresh cream | 19 | 18 | 18 | 9 | 18 | 6 |
|  | Oil | 11 | 11 | 11 | 5 | 11 | 1 |
| Mixed dishes | Cheese-based mixed dishes | 2 (1) | 0 | 0 | 0 | 1 | 0 |
|  | Pizzas, quiches and salty pastries | 40 | 25 | 40 | 14 | 40 | 5 |
|  | Fish and seafood mixed dishes | 10 | 0 | 10 | 3 | 10 | 1 |
|  | Meat-based mixed dishes | 23 (1) | 0 | 22 | 7 | 22 | 4 |
|  | Mixed salads | 15 (1) | 0 | 0 | 0 | 13 | 4 |
|  | Sandwiches | 50 (1) | 0 | 38 | 11 | 38 | 5 |
| Dairy | Milk-based desserts | 25 | 25 | 25 | 12 | 25 | 4 |
|  | Cottage cheese and similar | 18 | 18 | 18 | 8 | 18 | 5 |
|  | Ripened cheese (cooked-pressed, blue-veined, soft) | 57 (1) | 22 | 23 | 11 | 23 | 2 |
|  | Unripened, melted cheese | 21 | 21 | 21 | 8 | 21 | 1 |
|  | Milk | 13 | 11 | 11 | 6 | 11 | 4 |
|  | Milk powder | 3 | 0 | 0 | 0 | 0 | 0 |
|  | Yogurts and similar products | 28 | 28 | 28 | 13 | 28 | 5 |
| Salty sauces and condiments | Condiments | 15 | 4 | 0 | 0 | 0 | 0 |
|  | Herbs and spices | 12 | 0 | 0 | 0 | 0 | 0 |
|  | Cooked sauces (e.g. wine sauce, cheese sauce, butter sauce) | 33 (1) | 26 | 26 | 9 | 26 | 5 |
|  | Dipping sauces | 12 (1) | 11 | 11 | 5 | 11 | 2 |
|  | Salad dressing | 6 | 5 | 5 | 4 | 5 | 2 |
| Meat, fish and eggs | Offal | 33 (1) | 23 | 23 | 7 | 23 | 6 |
|  | Meat based main dishes | 38 (1) | 31 | 34 | 11 | 34 | 5 |
|  | Meat, unprocessed | 85 | 81 | 81 | 36 | 81 | 9 |
|  | Meat, cold cuts | 27 (1) | 12 | 12 | 5 | 12 | 2 |
|  | Crustaceans and mollusks, unprocessed | 24 (1) | 21 | 21 | 7 | 21 | 3 |
|  | Eggs and egg products | 13 (1) | 8 | 9 | 4 | 9 | 2 |
|  | Pâté, terrines, rillettes and foie gras | 15 | 0 | 0 | 0 | 0 | 0 |
|  | Fish, unprocessed | 44 | 44 | 44 | 16 | 44 | 3 |
|  | Fish and seafood based products | 29 (1) | 12 | 12 | 4 | 12 | 2 |
|  | Fish and seafood based main dishes | 21 (1) | 18 | 20 | 7 | 20 | 4 |
|  | Sausages | 16 | 16 | 16 | 8 | 16 | 2 |
| Foods for special nutritional use | Infant foods | 8 | 0 | 0 | 0 | 0 | 0 |
|  | Foods for special nutritional use (other than infant foods) | 10 | 0 | 0 | 0 | 0 | 0 |
|  | Total number of food items | 1444 | 1024 | 1124 | 478 | 1138 | 200 |

^1^ The number shown in parentheses corresponds to the number of food items in the corresponding subgroup which is an “average” food specifically created for the substitution of food items whose consumption is not recommended during pregnancy.

**^2^** Food items from the following subgroup were not considered for dietary changes: foods for particular nutritional use (n=18), alcoholic beverages (n=36), waters (n=32), hot beverages (tea, coffee, or cocoa) (n=20), flours (n=4), spices and herbs (n=12) and milk powders (n=3). Food items used as ingredients (e.g. “flaky pastry” in the “Cakes and pastries” subgroup) were also excluded. As the 134 food items not recommended during pregnancy had already been substituted in step 2, they could not be considered for any type of dietary change.

^3^All food items that are potential substitutes under type-3 dietary changes are “average” foods. They are not included in the count of total food items.
