# Supplementary material for: A clear trade-off exists between the theoretical efficiency and acceptability of dietary changes that improve nutrient adequacy during early pregnancy in French women: Combined data from simulated changes modeling and online assessment survey
Source: PLoS One. 2018 Apr 11;13(4):e0194764. doi: 10.1371/journal.pone.0194764 (PMC5895017; doi:10.1371/journal.pone.0194764)
Supplement: S4 Table — (DOCX) [file pone.0194764.s004.docx]

**S4 Table.** Characteristics of pregnant women (*n*=115) included in the acceptability study of dietary changes during pregnancy

|  | Total (*n*=115) | Group 1 (*n*=39) | Group 2 (*n*=38) | Group 3 (*n*=38) |
| --- | --- | --- | --- | --- |
| Age^1,4^ (years) | 31.1 ± 4.2 | 32.0 ± 4.2 | 31.2 ± 4.4 | 30.2 ± 4.1 |
| Months of pregnancy^2,5^  Less than 3  3 or 4  5 or 6 | 39.1% (45)  32.2% (37)  28.7% (33) | 35.9% (14)  33.3% (13)  30.8% (12) | 36.8% (14)  28.9% (11)  34.2% (13) | 44.7% (17)  34.2% (13)  21.1% (8) |
| Primiparous^2,5^ | 47.8% (55) | 41.0% (16) | 55.3% (21) | 47.4% (18) |
| Number of people composing the household^2,5^  1  2  3  4  5  6 or more | 0.9% (1)  52.2% (60)  36.5% (42)  7.0% (8)  1.7% (2)  1.7% (2) | 0% (0)  51.3% (20)  38.5% (15)  2.6% (1)  5.1% (2)  2.6% (1) | 2.6% (1)  52.6% (20)  34.2% (13)  10.5% (4)  0.0% (0)  0.0% (0) | 0.0% (0)  52.6% (20)  36.8% (14)  7.9% (3)  0.0% (0)  2.6% (1) |
| Number of children^2,5^  0  1  2  3  4 or more  Absence of answer | 47.8% (55)  40.0% (47)  7.8% (9)  1.7% (2)  1.7% (2)  0.9% (1) | 41.0% (16)  46.2% (18)  5.1% (2)  5.1% (2)  2.6% (1)  0.0% (0) | 52.6% (20)  34.2% (13)  10.5% (4)  0.0% (0)  0.0% (0)  2.6% (1) | 50.0% (19)  39.5% (15)  7.9% (3)  0.0% (0)  2.6% (1)  0.0% (0) |
| Occupation^2^  Farmer, Craftsperson, Storekeeper  Professional, executive  Intermediate profession  Employee  Manual worker  Student  Inactive | 0.9% (1)  20.0 % (23)  14.8% (17)  50.4% (58)  0.0% (0)  0.9% (1)  13.1% (15) | 0.0% (0)  15.4% (6)  20.5% (8)  51.3% (20)  0.0% (0)  0.0% (0)  12.8% (5) | 0.0% (0)  23.7% (9)  13.2% (5)  55.3% (21)  0.0% (0)  2.6% (1)  5.3% (2) | 2.6% (1)  21.1% (8)  10.5% (4)  44.7% (17)  0.0% (0)  0.0% (0)  21.1% (8) |
| Socio-professional category^2,3,5^  High  Low  Unemployed | 35.7% (41)  50.4% (58)  13.9% (16) | 35.9% (14)  51.3% (20)  12.8% (5) | 36.8% (14)  55.3% (21)  7.9% (3) | 34.2% (13)  44.7% (17)  21.1% (8) |
| Urbanization of the place of residence^2,5^  Paris  Major city (>100,000 inhab.)  Medium-sized town (20 – 100,000 inhab.)  Small-sized town (2 – 20,000 inhab.)  Rural area  Absence of answer | 8.7% (10)  26.1% (30)  24.3% (28)  25.2% (29)  13.0% (15)  2.6% (3) | 7.7% (3)  33.3% (13)  23.1% (9)  17.9% (7)  15.4% (6)  2.6% (1) | 13.2% (5)  23.7% (5)  18.2% (7)  26.3% (10)  18.4% (7)  0.0% (0) | 5.3% (2)  21.1% (8)  31.6% (12)  31.6% (12)  5.3% (2)  5.3% (2) |
| Nutrition awareness during pregnancy^2,5^  Much more aware  A little more aware  Not really more aware  Not more aware at all | 25.2% (29)  59.1% (68)  14.8% (17)  0.9% (1) | 23.1% (9)  56.4% (22)  20.5% (8)  0.0% (0) | 23.7% (9)  60.5% (23)  13.2% (5)  2.6% (1) | 28.9% (11)  60.5% (23)  10.5% (4)  0.0% (0) |

^1^ Values are mean ± SD

^2^ Values correspond to the percentage of participants presenting the characteristic described in the first column followed by the associated number of participants in parentheses.

^3^ Socio-professional categories were derived from occupations. “Farmer, craftsperson, storekeeper”, “Professional, executive”, and “Intermediate profession” belong to the High socio-professional category, “Employee”, “Manual worker” and “Student” belong to the Low socio-professional category and “Unemployed” belong to the Inactive socio-professional category.

^4^ No significant difference between groups for age as tested with a simple linear model, *P*>0.05

^5^ No significant difference between groups for the repartition of participants as tested with exact Fisher tests, *P*>0.05
