# Supplementary material for: A clear trade-off exists between the theoretical efficiency and acceptability of dietary changes that improve nutrient adequacy during early pregnancy in French women: Combined data from simulated changes modeling and online assessment survey
Source: PLoS One. 2018 Apr 11;13(4):e0194764. doi: 10.1371/journal.pone.0194764 (PMC5895017; doi:10.1371/journal.pone.0194764)
Supplement: S5 Table — (DOCX) [file pone.0194764.s005.docx]

**S5 Table.** PANDiet scores, Adeq-S, Mod-S, probabilities of adequacy for nutrient intakes and total energy intake excluding alcohol for the initial observed diet (D0), the initial observed modified diet (D0’), their delta (Δ0’) and the percentage of individuals with an increase between D0’ and D0 for women of childbearing age (*n*=344) participating in the ENNS^1^ study.

|  | Initial diet (D0)^2^ | Initial modified diet (D0’)^2^ | Delta (Δ0’)^3^ | *P*^4^ | Individuals with an increase (%) | *P*^5^ |
| --- | --- | --- | --- | --- | --- | --- |
| Energy intake excluding alcohol (kcal/d) | 1878.6 ± 402.0 | 1860.9 ± 394.9 | -17.66 ± 2.39  (-1.08) | <0.0001 | 33.4% | <0.0001 |
| PANDiet | 56.5 ± 7.4 | 57.1 ± 7.4 | + 0.65 ± 0.08  (0.16) | <0.0001 | 59.6% | <0.0001 |
| Adeq-S | 55.8 ± 12.7 | 55.6 ± 12.7 | - 0.20 ± 0.09  (0)^6^ | 0.03 | 35.8% | 0.008 |
| Protein | 0.97 ± 0.08 | 0.97 ± 0.08 | + 0.0013 ± 0.0003  (0)^6^ | <0.0001 | 47.1% | 0.007 |
| Total carbohydrate | 0.38 ± 0.39 | 0.40 ± 0.39 | + 0.018 ± 0.004  (0)^6^ | <0.0001 | 48.8% | 0.008 |
| Total fat | 0.92 ± 0.18 | 0.91 ± 0.19 | - 0.010 ± 0.002  (0)^6^ | <0.0001 | 31.7% | 0.004 |
| LA | 0.58 ± 0.34 | 0.56 ± 0.34 | - 0.016 ± 0.003  (0)^6^ | <0.0001 | 27.9% | <0.0001 |
| ALA | 0.08 ± 0.19 | 0.08 ± 0.18 | - 0.0061 ± 0.003  (0)^6^ | 0.02 | 42.7% | 0.95 |
| DHA | 0.17 ± 0.29 | 0.18 ± 0.29 | + 0.0065 ± 0.007  (0)^6^ | 0.32 | 47.7% | 0.04 |
| EPA + DHA | 0.15 ± 0.27 | 0.14 ± 0.26 | - 0.012 ± 0.007  (0)^6^ | 0.11 | 41.0% | 0.60 |
| Dietary fiber | 0.12 ± 0.20 | 0.12 ± 0.20 | - 0.00077 ± 0.0003  (0)^6^ | 0.004 | 67.7% | <0.0001 |
| Vitamin A | 0.78 ± 0.27 | 0.75 ± 0.29 | - 0.030 ± 0.007  (0)^6^ | <0.0001 | 50.6% | 0.001 |
| Thiamin | 0.27 ± 0.30 | 0.29 ± 0.31 | + 0.024 ± 0.004  (0)^6^ | <0.0001 | 52.6% | <0.0001 |
| Riboflavin | 0.80 ± 0.26 | 0.77 ± 0.28 | - 0.029 ± 0.004  (0)^6^ | <0.0001 | 19.5% | <0.0001 |
| Niacin | 0.73 ± 0.28 | 0.73 ± 0.28 | - 0.00072 ± 0.004  (0)^6^ | 0.85 | 32.3% | <0.0001 |
| Pantothenic acid | 0.64 ± 0.32 | 0.63 ± 0.32 | - 0.0062 ± 0.002  (0)^6^ | 0.005 | 39.8% | <0.0001 |
| Vitamin B6 | 0.37 ± 0.35 | 0.36 ± 0.35 | - 0.0050 ± 0.003  (0)^6^ | 0.07 | 29.7% | <0.0001 |
| Folate | 0.52 ± 0.32 | 0.46 ± 0.32 | - 0.051 ± 0.005  (-0.018) | <0.0001 | 18.0% | <0.0001 |
| Vitamin B12 | 0.88 ± 0.20 | 0.87 ± 0.21 | - 0.012 ± 0.004  (0)^6^ | 0.002 | 38.4% | 0.21 |
| Vitamin C | 0.43 ± 0.38 | 0.43 ± 0.38 | + 0.0027 ± 0.0008  (0)^6^ | 0.0011 | 64.8% | <0.0001 |
| Vitamin D | 0.03 ± 0.12 | 0.03 ± 0.11 | - 0.0027 ± 0.002  (0)^6^ | 0.25 | 41.3% | 0.68 |
| Vitamin E | 0.54 ± 0.34 | 0.54 ± 0.34 | + 0.0010 ± 0.002  (0)^6^ | 0.66 | 40.4% | 0.45 |
| Calcium | 0.75 ± 0.29 | 0.78 ± 0.28 | + 0.036 ± 0.004  (0)^6^ | <0.0001 | 64.5% | <0.0001 |
| Iron | 0.77 ± 0.18 | 0.76 ± 0.19 | - 0.015 ± 0.003  (0)^6^ | <0.0001 | 2.3% | <0.0001 |
| Iodine | 0.22 ± 0.24 | 0.22 ± 0.24 | + 0.0010 ± 0.001  (0)^6^ | 0.31 | 47.4% | 0.05 |
| Magnesium | 0.38 ± 0.36 | 0.39 ± 0.36 | + 0.012 ± 0.001  (0)^6^ | <0.0001 | 72.7% | <0.0001 |
| Phosphorus | 0.98 ± 0.06 | 0.98 ± 0.05 | + 0.0019 ± 0.0004  (0)^6^ | <0.0001 | 57.3% | <0.0001 |
| Potassium | 0.64 ± 0.31 | 0.65 ± 0.31 | + 0.0013 ± 0.001  (0)^6^ | 0.22 | 39.5% | 0.27 |
| Selenium | 0.68 ± 0.31 | 0.70 ± 0.30 | + 0.022 ± 0.004  (0)^6^ | <0.0001 | 62.2% | <0.0001 |
| Zinc | 0.89 ± 0.18 | 0.90 ± 0.16 | + 0.013 ± 0.002  (0)^6^ | <0.0001 | 66.3% | <0.0001 |
| Mod-S | 57.2 ± 11.8 | 58.7 ± 11.6 | + 1.51 ± 0.17  (0.37) | <0.0001 | 64.8% | <0.0001 |
| Protein | 0.97 ± 0.11 | 0.97 ± 0.12 | - 0.0052 ± 0.002  (0)^6^ | 0.008 | 16.0% | <0.0001 |
| Total carbohydrate | 0.99 ± 0.07 | 0.99 ± 0.06 | + 0.00076 ± 0.002  (0)^6^ | 0.62 | 26.7% | 0.16 |
| Free sugars | 0.54 ± 0.38 | 0.54 ± 0.39 | - 0.0064 ± 0.002  (0)^6^) | <0.0001 | 35.5% | 0.007 |
| Total fat | 0.55 ± 0.39 | 0.59 ± 0.38 | + 0.039 ± 0.007  (0)^6^ | <0.0001 | 56.7% | <0.0001 |
| SFA | 0.14 ± 0.21 | 0.15 ± 0.22 | + 0.014 ± 0.002  (0)^6^ | <0.0001 | 59.6% | <0.0001 |
| Cholesterol | 0.45 ± 0.34 | 0.49 ± 0.34 | + 0.035 ± 0.005  (0)^6^ | <0.0001 | 58.7% | <0.0001 |
| Sodium | 0.36 ± 0.31 | 0.39 ± 0.31 | + 0.027 ± 0.004  (0)^6^ | <0.0001 | 62.8% | <0.0001 |
| Penalty | -0.01 ± 0.11 | -0.01 ± 0.09 | + 0.0029 ± 0.007  (0)^6^ | 0.66 | 0.9% | 1.00 |

^1^ *Etude Nationale Nutrition Santé*, 2006-2007.

^2^ Values are mean ± SD

^3^ Values are mean ± SEM

^4^ Student t-tests with a Bonferroni correction were performed to define whether the means of the delta for PANDiet scores, Adeq-S, Mod-S, probabilities of adequacy for nutrient intakes and energy intake excluding alcohol between the initial observed diet (D0) and the initial observed modified diet (D0’), named Δ0’,were different from 0.

^5^ Sign tests with a Bonferroni correction were performed to define whether the percentage of individuals with a positive or negative Δ0’ for PANDiet score, Adeq-S, Mod-S, probabilities of adequacy for nutrient intakes and energy intake excluding alcohol were different.

^6^ Median is between -0.01 and 0.01.

Adeq-S, Adequacy sub-score of the PANDiet. ALA, alpha linolenic Acid. DHA, docosahexaenoic acid. EPA, eicosapentaenoic acid. LA, linoleic acid. Mod-S, Moderation sub-score of the PANDiet. SFA, saturated fatty acids.
