# Supplementary material for: A clear trade-off exists between the theoretical efficiency and acceptability of dietary changes that improve nutrient adequacy during early pregnancy in French women: Combined data from simulated changes modeling and online assessment survey
Source: PLoS One. 2018 Apr 11;13(4):e0194764. doi: 10.1371/journal.pone.0194764 (PMC5895017; doi:10.1371/journal.pone.0194764)
Supplement: S6 Table — (DOCX) [file pone.0194764.s006.docx]

**S6 Table.** PANDiet score, Adeq-S, Mod-S, total energy intake without alcohol and probabilities of adequacy for nutrient intakes for D0’ (initial observed modified diet) and their changes in final simulated diets under type-1 (Δ1), type-2 (Δ2) and type-3 (Δ3) changes for women of childbearing age (*n*=344) participating in the ENNS^1^ study.

|  | | Delta between the initial observed modified diet (D0’) and... | | |  |
| --- | --- | --- | --- | --- | --- |
|  | Initial observed modified diet (D0’)^2^ | Final simulated diet under type-1changes  (Δ1)^3^ | Final simulated diet under type-2 changes  (Δ2) ^3^ | Final simulated diet under type-3 changes  (Δ3) ^3^ | *P* ^4^ |
| Energy intake without alcohol (kcal/d) | 1860.9 ± 394.9 | + 94.0 ± 2.65^a^ | + 32.3 ± 1.94^b^ | + 49.5 ± 2.42^c^ | **<0.001** |
| PANDiet score | 57.1 ± 7.4 | + 9.78 ± 0.18^a^ | + 14.84 ± 0.20^b^ | + 23.93 ± 0.26^c^ | **<0.001** |
| Adeq-S | 55.6 ± 12.7 | + 9.29 ± 0.26^a^ | + 14.10 ± 0.30^b^ | + 23.24 ± 0.37^c^ | **<0.001** |
| Protein | 0.97 ± 0.08 | + 0.014 ± 0.0029^a.b^ | + 0.0088 ± 0.002^b^ | + 0.018 ± 0.0034^a^ | **0.027** |
| Total carbohydrate | 0.40 ± 0.39 | + 0.30 ± 0.018^a^ | + 0.20 ± 0.015^b^ | + 0.27 ± 0.021^a^ | **<0.001** |
| Total fat | 0.91 ± 0.19 | - 0.031 ± 0.0066^a^ | + 0.014 ± 0.0092^b^ | + 0.044 ± 0.010^b^ | **<0.001** |
| LA | 0.56 ± 0.34 | + 0.060 ± 0.0090^a^ | + 0.29 ± 0.016^b^ | + 0.38 ± 0.018^c^ | **<0.001** |
| ALA | 0.08 ± 0.18 | + 0.013 ± 0.0049^a^ | + 0.23 ± 0.015^b^ | + 0.41 ± 0.017^c^ | **<0.001** |
| DHA | 0.18 ± 0.29 | + 0.055 ± 0.0082^a^ | + 0.14 ± 0.014^b^ | + 0.70 ± 0.018^c^ | **<0.001** |
| EPA + DHA | 0.14 ± 0.26 | + 0.054 ± 0.0080^a^ | + 0.15 ± 0.014^b^ | + 0.71 ± 0.017^c^ | **<0.001** |
| Dietary fiber | 0.12 ± 0.20 | + 0.13 ± 0.0080^a^ | + 0.080 ± 0.0067^b^ | + 0.24 ± 0.012^c^ | **<0.001** |
| Vitamin A | 0.75 ± 0.29 | + 0.0082 ± 0.0081^a^ | + 0.093 ± 0.012^b^ | - 0.086 ± 0.014^c^ | **<0.001** |
| Thiamin | 0.29 ± 0.31 | + 0.13 ± 0.0077^a^ | + 0.22 ± 0.014^b^ | + 0.41 ± 0.015^c^ | **<0.001** |
| Riboflavin | 0.77 ± 0.28 | + 0.077 ± 0.0064^a^ | + 0.082 ± 0.0084^a^ | + 0.16 ± 0.013^b^ | **<0.001** |
| Niacin | 0.73 ± 0.28 | + 0.13 ± 0.0084^a^ | + 0.16 ± 0.011^a^ | + 0.25 ± 0.015^b^ | **<0.001** |
| Pantothenic acid | 0.63 ± 0.32 | + 0.15 ± 0.0080^a^ | + 0.10 ± 0.0093^b^ | + 0.29 ± 0.014^c^ | **<0.001** |
| Vitamin B6 | 0.36 ± 0.35 | + 0.20 ± 0.011^a^ | + 0.28 ± 0.013^b^ | + 0.58 ± 0.018^c^ | **<0.001** |
| Folate | 0.46 ± 0.32 | + 0.15 ± 0.0078^a^ | + 0.26 ± 0.011^b^ | + 0.26 ± 0.012^b^ | **<0.001** |
| Vitamin B12 | 0.87 ± 0.21 | + 0.023 ± 0.0058^a^ | + 0.045 ± 0.0084^a^ | + 0.10 ± 0.011^b^ | **<0.001** |
| Vitamin C | 0.43 ± 0.38 | + 0.19 ± 0.012^a^ | + 0.26 ± 0.017^b^ | + 0.38 ± 0.017^c^ | **<0.001** |
| Vitamin D | 0.03 ± 0.11 | + 0.0078 ± 0.0027^a^ | + 0.025 ± 0.0057^a^ | + 0.42 ± 0.017^b^ | **<0.001** |
| Vitamin E | 0.54 ± 0.34 | + 0.15 ± 0.0099^a^ | + 0.31 ± 0.015^b^ | + 0.35 ± 0.015^c^ | **<0.001** |
| Calcium | 0.78 ± 0.28 | + 0.051 ± 0.0074^a^ | + 0.066 ± 0.0071^a^ | - 0.12 ± 0.011^b^ | **<0.001** |
| Iron | 0.76 ± 0.19 | + 0.076 ± 0.0045^a^ | + 0.13 ± 0.0072^b^ | + 0.15 ± 0.0085^c^ | **<0.001** |
| Iodine | 0.22 ± 0.24 | + 0.040 ± 0.0063^a^ | + 0.039 ± 0.011^a^ | + 0.074 ± 0.011^a^ | **0.30** |
| Magnesium | 0.39 ± 0.36 | + 0.16 ± 0.0085^a^ | + 0.25 ± 0.013^b^ | + 0.30 ± 0.013^c^ | **<0.001** |
| Phosphorus | 0.98 ± 0.05 | + 0.0093 ± 0.0021^a.b^ | + 0.0068 ± 0.0014^a^ | + 0.012 ± 0.0026^b^ | **0.031** |
| Potassium | 0.65 ± 0.31 | + 0.18 ± 0.0089^a^ | + 0.15 ± 0.0081^b^ | + 0.22 ± 0.011^c^ | **<0.001** |
| Selenium | 0.70 ± 0.30 | + 0.082 ± 0.0070^a^ | + 0.20 ± 0.014^b^ | + 0.23 ± 0.014^b^ | **<0.001** |
| Zinc | 0.90 ± 0.16 | + 0.041 ± 0.0045^a^ | + 0.039 ± 0.0058^a^ | - 0.013 ± 0.0074^b^ | **<0.001** |
| Mod-S | 58.7 ± 11.6 | + 10.28 ± 0.33^a^ | + 15.58 ± 0.42^b^ | + 24.61 ± 0.47^c^ | **<0.001** |
| Protein | 0.97 ± 0.12 | + 0.018 ± 0.0041^a^ | + 0.012 ± 0.0037^a^ | + 0.014 ± 0.0041^a^ | 1.00 |
| Total carbohydrate | 0.99 ± 0.06 | + 0.0069 ± 0.0027^a^ | + 0.0082 ± 0.0028^a.b^ | + 0.0095 ± 0.0030^b^ | 0.078 |
| Free sugars | 0.54 ± 0.39 | + 0.16 ± 0.012^a^ | + 0.19 ± 0.014^a^ | + 0.25 ± 0.016^b^ | **<0.001** |
| Total fat | 0.59 ± 0.38 | + 0.27 ± 0.016^a^ | + 0.24 ± 0.016^a^ | + 0.32 ± 0.018^b^ | **<0.001** |
| SFA | 0.15 ± 0.22 | + 0.18 ± 0.0094^a^ | + 0.31 ± 0.013^b^ | + 0.53 ± 0.014^c^ | **<0.001** |
| Cholesterol | 0.49 ± 0.34 | + 0.14 ± 0.0090^a^ | + 0.25 ± 0.013^b^ | + 0.39 ± 0.016^c^ | **<0.001** |
| Sodium | 0.39 ± 0.31 | -0.052 ± 0.0066^a^ | + 0.074 ± 0.0075^b^ | + 0.21 ± 0.012^c^ | **<0.001** |
| Penalty | 0.01 ± 0.09 | -0.0058 ± 0.0041^a^ | + 0.01 ± 0.009^a^ | + 0.01 ± 0.009^a^ | 1.00 |

^1^ *Etude Nationale Nutrition Santé*, 2006-2007.

^2^ Values are mean ± SD

^3^ Values are mean ± SEM

^4^ The effects of the type of dietary changes were assessed under a mixed model with a random effect on the individual where the dependent variable was the delta for the variables presented in the first column, and the independent variable was the type of dietary changes. ^a,b,c^ Comparisons of means between Δ1, Δ2 and Δ3 were performed under this model with a Bonferroni correction. *P*<0.05

Adeq-S, Adequacy sub-score of the PANDiet. ALA, alpha linolenic acid. DHA, docosahexaenoic acid. EPA, eicosapentaenoic acid. LA, linoleic acid. Mod-S, Moderation sub-score of the PANDiet. SFA, saturated fatty acids.
