# Supplementary material for: A clear trade-off exists between the theoretical efficiency and acceptability of dietary changes that improve nutrient adequacy during early pregnancy in French women: Combined data from simulated changes modeling and online assessment survey
Source: PLoS One. 2018 Apr 11;13(4):e0194764. doi: 10.1371/journal.pone.0194764 (PMC5895017; doi:10.1371/journal.pone.0194764)
Supplement: S7 Table — (DOCX) [file pone.0194764.s007.docx]

**S7 Table.** PANDiet scores, Adeq-S, Mod-S, probabilities of adequacy for nutrient intakes and total energy intake excluding alcohol for the initial observed modified diet (D0’), the final simulated diet under type-1 dietary changes (D1), their delta (Δ1) and the percentage of individuals with an increase between D1 and D0’ for women of childbearing age (*n*=344) participating in the ENNS^1^ study.

|  | Initial observed modified diet (D0’)^2^ | Final simulated diet under type-1 changes (D1)^2^ | Delta 1  (Δ1)^3^ | *P*^4^ | Individual with an increase | *P*^5^ |
| --- | --- | --- | --- | --- | --- | --- |
| Energy intake without alcohol (kcal/d) | 1860.9 ± 394.9 | 1954.9 ± 384.5 | + 94.0 ± 2.65  (109.4) | <0.0001 | 100% | <0.0001 |
| PANDiet | 57.1 ± 7.4 | 66.9 ± 7.3 | + 9.78 ± 0.18  (9.59) | <0.0001 | 100% | <0.0001 |
| Adeq-S | 55.6 ± 12.7 | 64.9 ± 10.7 | + 9.29 ± 0.26  (8.76) | <0.0001 | 98.8% | <0.0001 |
| Protein | 0.97 ± 0.08 | 0.99 ± 0.04 | + 0.014 ± 0.0029  (0)^6^ | <0.0001 | 68.0% | <0.0001 |
| Total carbohydrate | 0.40 ± 0.39 | 0.70 ± 0.36 | + 0.30 ± 0.018  (0.20) | <0.0001 | 81.4% | <0.0001 |
| Total fat | 0.91 ± 0.19 | 0.88 ± 0.21 | - 0.031 ± 0.0066  (0)^6^ | 0.00011 | 32.0% | <0.0001 |
| LA | 0.56 ± 0.34 | 0.62 ± 0.32 | + 0.060 ± 0.0090  (0)^6^ | <0.0001 | 55.8% | 1.00 |
| ALA | 0.08 ± 0.18 | 0.09 ± 0.20 | + 0.013 ± 0.0049  (0)^6^ | 0.26 | 38.7% | 0.0012 |
| DHA | 0.18 ± 0.29 | 0.24 ± 0.33 | + 0.055 ± 0.0082  (0)^6^ | <0.0001 | 45.3% | 1.00 |
| EPA + DHA | 0.14 ± 0.26 | 0.19 ± 0.29 | + 0.054 ± 0.0080  (0)^6^ | <0.0001 | 43.0% | 1.00 |
| Dietary fiber | 0.12 ± 0.20 | 0.25 ± 0.28 | + 0.13 ± 0.0080  (0.075) | <0.0001 | 97.7% | <0.0001 |
| Vitamin A | 0.75 ± 0.29 | 0.76 ± 0.28 | + 0.0082 ± 0.0081  (0)^6^ | 1.00 | 45.3% | 1.00 |
| Thiamin | 0.29 ± 0.31 | 0.42 ± 0.33 | + 0.13 ± 0.0077  (0.089) | <0.0001 | 91.9% | <0.0001 |
| Riboflavin | 0.77 ± 0.28 | 0.85 ± 0.23 | + 0.077 ± 0.0064  (0.026) | <0.0001 | 76.7% | <0.0001 |
| Niacin | 0.73 ± 0.28 | 0.86 ± 0.20 | + 0.13 ± 0.0084  (0.076) | <0.0001 | 91.6% | <0.0001 |
| Pantothenic acid | 0.63 ± 0.32 | 0.78 ± 0.25 | + 0.15 ± 0.0080  (0.11) | <0.0001 | 91.9% | <0.0001 |
| Vitamin B6 | 0.36 ± 0.35 | 0.57 ± 0.34 | + 0.20 ± 0.011  (0.17) | <0.0001 | 93.6% | <0.0001 |
| Folate | 0.46 ± 0.32 | 0.62 ± 0.30 | + 0.15 ± 0.0078  (0.12) | <0.0001 | 91.3% | <0.0001 |
| Vitamin B12 | 0.87 ± 0.21 | 0.89 ± 0.18 | + 0.023 ± 0.0058  (0)^6^ | 0.0023 | 51.2% | 1.00 |
| Vitamin C | 0.43 ± 0.38 | 0.62 ± 0.35 | + 0.19 ± 0.012  (0.097) | <0.0001 | 91.9% | <0.0001 |
| Vitamin D | 0.03 ± 0.11 | 0.04 ± 0.13 | + 0.0078 ± 0.0027  (0)^6^ | 0.13 | 30.8% | <0.0001 |
| Vitamin E | 0.54 ± 0.34 | 0.70 ± 0.30 | + 0.15 ± 0.0099  (0.098) | <0.0001 | 86.6% | <0.0001 |
| Calcium | 0.78 ± 0.28 | 0.84 ± 0.23 | + 0.051 ± 0.0074  (0)^6^ | <0.0001 | 66.3% | <0.0001 |
| Iron | 0.76 ± 0.19 | 0.83 ± 0.15 | + 0.076 ± 0.0045  (0.08) | <0.0001 | 64.5% | <0.0001 |
| Iodine | 0.22 ± 0.24 | 0.26 ± 0.27 | + 0.040 ± 0.0063  (0.011) | <0.0001 | 68.6% | <0.0001 |
| Magnesium | 0.39 ± 0.36 | 0.55 ± 0.37 | + 0.16 ± 0.0085  (0.12) | <0.0001 | 95.6% | <0.0001 |
| Phosphorus | 0.98 ± 0.05 | 0.99 ± 0.02 | + 0.0093 ± 0.0021  (0)^6^ | 0.00059 | 69.8% | <0.0001 |
| Potassium | 0.65 ± 0.31 | 0.83 ± 0.23 | + 0.18 ± 0.0089  (0.14) | <0.0001 | 95.3% | <0.0001 |
| Selenium | 0.70 ± 0.30 | 0.78 ± 0.26 | + 0.082 ± 0.0070  (0.038) | <0.0001 | 78.8% | <0.0001 |
| Zinc | 0.90 ± 0.16 | 0.94 ± 0.11 | + 0.041 ± 0.0045  (0)^6^ | <0.0001 | 75.0% | <0.0001 |
| Mod-S | 58.7 ± 11.6 | 69.0 ± 11.2 | + 10.28 ± 0.33 (10.06) | <0.0001 | 97.7% | <0.0001 |
| Protein | 0.97 ± 0.12 | 0.99 ± 0.07 | + 0.018 ± 0.0041  (0)^6^ | 0.00045 | 38.7% | 1.00 |
| Total carbohydrate | 0.99 ± 0.06 | 1.00 ± 0.01 | + 0.0069 ± 0.0027  (0)^6^ | 0.48 | 40.4% | 1.00 |
| Free sugars | 0.54 ± 0.39 | 0.70 ± 0.37 | + 0.16 ± 0.012  (0.047) | <0.0001 | 80.5% | <0.0001 |
| Total Fat | 0.59 ± 0.38 | 0.86 ± 0.28 | + 0.27 ± 0.016  (0.15) | <0.0001 | 86.0% | <0.0001 |
| SFA | 0.15 ± 0.22 | 0.33 ± 0.30 | + 0.18 ± 0.0094  (0.13) | <0.0001 | 95.3% | <0.0001 |
| Cholesterol | 0.49 ± 0.34 | 0.62 ± 0.34 | + 0.14 ± 0.0090  (0.084) | <0.0001 | 80.2% | <0.0001 |
| Sodium | 0.39 ± 0.31 | 0.34 ± 0.31 | -0.052 ± 0.0066  (-0.037) | <0.0001 | 22.1% | <0.0001 |
| Penalty | 0.01 ± 0.09 | 0.002 ± 0.05 | -0.0058 ± 0.0041  (0) | 1.00 | 0.6% | 1.00 |

^1^ *Etude Nationale Nutrition Santé*, 2006-2007.

^2^ Values are mean ± SD

^3^ Values are mean ± SEM

^4^ Student t-tests with a Bonferroni correction were performed to define whether the means of the delta for PANDiet scores, Adeq-S, Mod-S, probabilities of adequacy for nutrient intakes and energy intake excluding alcohol between the initial observed modified diet (D0’) and the final simulated diet under type-1 dietary changes, named Δ1, were different from 0.

^5^ Sign tests with a Bonferroni correction were performed to define whether the percentage of individuals with a positive or negative Δ1 for PANDiet scores, Adeq-S, Mod-S, probabilities of adequacy for nutrient intakes and energy intake excluding alcohol were different.

**^6^** Median is between -0.01 and 0.01.

Adeq-S, Adequacy sub-score of the PANDiet. ALA, alpha linolenic acid. DHA, docosahexaenoic acid. EPA, eicosapentaenoic acid. LA, linoleic acid. Mod-S, Moderation sub-score of the PANDiet. SFA, saturated fatty acids.
