# Supplementary material for: A clear trade-off exists between the theoretical efficiency and acceptability of dietary changes that improve nutrient adequacy during early pregnancy in French women: Combined data from simulated changes modeling and online assessment survey
Source: PLoS One. 2018 Apr 11;13(4):e0194764. doi: 10.1371/journal.pone.0194764 (PMC5895017; doi:10.1371/journal.pone.0194764)
Supplement: S9 Table — (DOCX) [file pone.0194764.s009.docx]

**S9 Table.** PANDiet scores, Adeq-S, Mod-S, probabilities of adequacy for nutrient intakes and total energy intake excluding alcohol for the initial observed modified diet (D0’), the final simulated diet under type-3 dietary changes (D3), their delta (Δ3) and the percentage of individuals with an increase between D3 and D0’ for women of childbearing age (n=344) participating in the ENNS^1^ study.

|  | Initial observed modified diet  (D0’)^2^ | Final simulated diet under type-3 changes (D3)^2^ | Delta 3  (Δ3)^3^ | *P*^4^ | Individual with an increase | *P*^5^ |
| --- | --- | --- | --- | --- | --- | --- |
| Energy intake without alcohol (kcal/d) | 1860.9 ± 394.9 | 1910.4 ± 385.7 | + 49.5 ± 2.42  (33.7) | <0.0001 | 100% | <0.0001 |
| PANDiet | 57.1 ± 7.4 | 81.6 ± 6.2 | + 23.93 ± 0.26  (23.68) | <0.0001 | 100% | <0.0001 |
| Adeq-S | 55.6 ± 12.7 | 78.8 ± 9.3 | + 23.24 ± 0.37  (23.06) | <0.0001 | 100% | <0.0001 |
| Protein | 0.97 ± 0.08 | 0.99 ± 0.03 | + 0.018 ± 0.0034  (0)^6^ | <0.0001 | 66.0% | <0.0001 |
| Total carbohydrate | 0.40 ± 0.39 | 0.67 ± 0.37 | + 0.27 ± 0.021  (0.16) | <0.0001 | 75.3% | <0.0001 |
| Total fat | 0.91 ± 0.19 | 0.96 ± 0.10 | + 0.044 ± 0.010  (0)^6^ | 0.00073 | 51.7% | 1.00 |
| LA | 0.56 ± 0.34 | 0.93 ± 0.12 | + 0.38 ± 0.018  (0.30) | <0.0001 | 91.8% | <0.0001 |
| ALA | 0.08 ± 0.18 | 0.49 ± 0.32 | + 0.41 ± 0.017  (0.41) | <0.0001 | 98.0% | <0.0001 |
| DHA | 0.18 ± 0.29 | 0.88 ± 0.19 | + 0.70 ± 0.018  (0.82) | <0.0001 | 96.2% | <0.0001 |
| EPA + DHA | 0.14 ± 0.26 | 0.85 ± 0.21 | + 0.71 ± 0.017  (0.82) | <0.0001 | 96.8% | <0.0001 |
| Dietary Fiber | 0.12 ± 0.20 | 0.36 ± 0.31 | + 0.24 ± 0.012  (0.20) | <0.0001 | 97.1% | <0.0001 |
| Vitamin A | 0.75 ± 0.29 | 0.66 ± 0.31 | - 0.086 ± 0.014  (-0.077) | <0.0001 | 23.8% | <0.0001 |
| Thiamin | 0.29 ± 0.31 | 0.70 ± 0.27 | + 0.41 ± 0.015  (0.40) | <0.0001 | 93.9% | <0.0001 |
| Riboflavin | 0.77 ± 0.28 | 0.93 ± 0.12 | + 0.16 ± 0.013  (0.043) | <0.0001 | 72.7% | <0.0001 |
| Niacin | 0.73 ± 0.28 | 0.98 ± 0.05 | + 0.25 ± 0.015  (0.14) | <0.0001 | 96.2% | <0.0001 |
| Pantothenic acid | 0.63 ± 0.32 | 0.92 ± 0.14 | + 0.29 ± 0.014  (0.22) | <0.0001 | 94.8% | <0.0001 |
| Vitamin B6 | 0.36 ± 0.35 | 0.94 ± 0.11 | + 0.58 ± 0.018  (0.66) | <0.0001 | 99.4% | <0.0001 |
| Folate | 0.46 ± 0.32 | 0.73 ± 0.26 | + 0.26 ± 0.012  (0.24) | <0.0001 | 92.7% | <0.0001 |
| Vitamin B12 | 0.87 ± 0.21 | 0.97 ± 0.05 | + 0.10 ± 0.011  (0.017) | <0.0001 | 75.6% | <0.0001 |
| Vitamin C | 0.43 ± 0.38 | 0.81 ± 0.25 | + 0.38 ± 0.017  (0.38) | <0.0001 | 90.4% | <0.0001 |
| Vitamin D | 0.03 ± 0.11 | 0.45 ± 0.32 | + 0.42 ± 0.017  (0.42) | <0.0001 | 95.1% | <0.0001 |
| Vitamin E | 0.54 ± 0.34 | 0.90 ± 0.15 | + 0.35 ± 0.015  (0.31) | <0.0001 | 97.7% | <0.0001 |
| Calcium | 0.78 ± 0.28 | 0.66 ± 0.30 | - 0.12 ± 0.011  (-0.072) | <0.0001 | 20.6% | <0.0001 |
| Iron | 0.76 ± 0.19 | 0.91 ± 0.08 | + 0.15 ± 0.0085  (0.10) | <0.0001 | 82.3% | <0.0001 |
| Iodine | 0.22 ± 0.24 | 0.29 ± 0.27 | + 0.074 ± 0.011  (0.024) | <0.0001 | 67.4% | <0.0001 |
| Magnesium | 0.39 ± 0.36 | 0.69 ± 0.33 | + 0.30 ± 0.013  (0.27) | <0.0001 | 98.3% | <0.0001 |
| Phosphorus | 0.98 ± 0.05 | 1.00 ± 0.01 | + 0.012 ± 0.0026  (0)^6^ | 0.00015 | 73.0% | <0.0001 |
| Potassium | 0.65 ± 0.31 | 0.87 ± 0.19 | + 0.22 ± 0.011  (0.18) | <0.0001 | 96.5% | <0.0001 |
| Selenium | 0.70 ± 0.30 | 0.93 ± 0.11 | + 0.23 ± 0.014  (0.12) | <0.0001 | 85.5% | <0.0001 |
| Zinc | 0.90 ± 0.16 | 0.89 ± 0.14 | - 0.013 ± 0.0074  (0)^6^ | 1.00 | 33.7% | <0.0001 |
| Mod-S | 58.7 ± 11.6 | 83.3 ± 10.1 | + 24.61 ± 0.47  (23.87) | <0.0001 | 100% | <0.0001 |
| Protein | 0.97 ± 0.12 | 0.98 ± 0.08 | + 0.014 ± 0.0041  (0)^6^ | 0.022 | 35.8% | 1.00 |
| Total carbohydrate | 0.99 ± 0.06 | 1.00 ± 0.01 | + 0.0095 ± 0.0030  (0)^6^ | 0.061 | 46.8% | 1.00 |
| Free sugars | 0.54 ± 0.39 | 0.79 ± 0.32 | + 0.25 ± 0.016  (0.13) | <0.0001 | 75.9% | <0.0001 |
| Total Fat | 0.59 ± 0.38 | 0.91 ± 0.23 | + 0.32 ± 0.018  (0.22) | <0.0001 | 84.3% | <0.0001 |
| SFA | 0.15 ± 0.22 | 0.68 ± 0.29 | + 0.53 ± 0.014  (0.58) | <0.0001 | 98.8% | <0.0001 |
| Cholesterol | 0.49 ± 0.34 | 0.88 ± 0.20 | + 0.39 ± 0.016  (0.35) | <0.0001 | 95.9% | <0.0001 |
| Sodium | 0.39 ± 0.31 | 0.60 ± 0.33 | + 0.21 ± 0.012  (0.18) | <0.0001 | 79.7% | <0.0001 |
| Penalty | -0.01 ± 0.09 | 0 | + 0.01 ± 0.009  (0) | 1.00 | 0.9% | 1.00 |

^1^ *Etude Nationale Nutrition Santé*, 2006-2007.

^2^ Values are mean ± SD

^3^ Values are mean ± SEM

^4^ Student t-tests with a Bonferroni correction were performed to define whether the means of the delta for PANDiet scores, Adeq-S, Mod-S, probabilities of adequacy for nutrient intakes and energy intake excluding alcohol between the initial observed modified diet (D0’) and the final simulated diet under type-3 dietary changes (D3), named Δ3, were different from 0.

^5^ Sign tests with a Bonferroni correction were performed to define whether the percentage of individuals with a positive or negative Δ3 for PANDiet scores, Adeq-S, Mod-S, probabilities of adequacy for nutrient intakes and energy intake excluding alcohol were different.

**^6^** Median is between -0.01 and 0.01.

Adeq-S, Adequacy sub-score of the PANDiet. ALA, alpha linolenic acid. DHA, docosahexaenoic acid. EPA, eicosapentaenoic acid. LA, linoleic acid. Mod-S, Moderation sub-score of the PANDiet. SFA, saturated fatty acids.
