# Supplementary material for: A clear trade-off exists between the theoretical efficiency and acceptability of dietary changes that improve nutrient adequacy during early pregnancy in French women: Combined data from simulated changes modeling and online assessment survey
Source: PLoS One. 2018 Apr 11;13(4):e0194764. doi: 10.1371/journal.pone.0194764 (PMC5895017; doi:10.1371/journal.pone.0194764)
Supplement: S1 Text — (DOCX) [file pone.0194764.s012.docx]

**S1 Text.** Details of each sub-score composing the PANDiet score.

In the updated version, each sub-score is composed of probabilities of adequacy for nutrients (27 for the Adeq-S: protein, total carbohydrate, dietary fiber, total fat, LA, ALA, DHA, EPA+DHA, vitamin A, thiamin, riboflavin, niacin, pantothenic acid, vitamin B6, folate, vitamin B12, vitamin C, vitamin D, vitamin E, calcium, iodine, iron, magnesium, phosphorus, potassium, selenium and zinc and 7 for the Mod-S: protein, total carbohydrate, free sugars, total fat, saturated fatty acids, cholesterol and sodium), with a further 14 potential penalties for exceeding the tolerable upper intake limits (retinol, niacin, vitamin B6, folate, vitamin C, vitamin D, vitamin E, calcium, iodine, iron, magnesium, phosphorus, selenium and zinc) which are added to the Mod-S. As DHA intakes are considered in the probabilities of adequacy for both DHA and EPA+DHA intakes, in this study, each one was weighted by 0.5 in the final score, which resulted in an Adeq-S with 26 probabilities of adequacy for nutrient intakes.
