# Supplementary material for: A clear trade-off exists between the theoretical efficiency and acceptability of dietary changes that improve nutrient adequacy during early pregnancy in French women: Combined data from simulated changes modeling and online assessment survey
Source: PLoS One. 2018 Apr 11;13(4):e0194764. doi: 10.1371/journal.pone.0194764 (PMC5895017; doi:10.1371/journal.pone.0194764)
Supplement: S2 Text — (DOCX) [file pone.0194764.s013.docx]

**S2 Text.** Details about the three types of dietary changes used in the simulation study.

*Type-1: modification to the amount consumed of a food item*

The first type of dietary change related to modifying the amount of a food item present in the observed diet (D0’). To be considered for type-1, a food item should not contain too many ingredients or foods at unidentifiable proportions (*i.e.* all items belonging to the mixed dishes group, except for some pizzas and quiches with quite simple and constant recipes) and should be consumed by the population to identify the amounts usually consumed. For the food items considered, this amount could be increased or decreased in a stepped manner. A step size was attributed to each food item according to the observed consumption of participants in the ENNS survey (adults, non under-reporters, *n*=2756).The step size was thus defined for 1146 food items according to the process described below.

To define the step size, 1146 food items were considered. Food items were gathered in “serving size subgroups” (n=241), which corresponded to a set of food items where the same amount is generally consumed on the same moment of consumption. Eighty-five “serving size subgroups” were defined for the 194 food items sold by unit or pack in stores (*e.g.* a cereal bar, a yogurt), then the step was defined as the quantity contained in one unit or one pack. For each of the other 156 “serving size subgroups”, *i*, (corresponding to 952 food items), the step, STEP*i*, was defined as the following amount rounded to 5 grams:

$$STEPi= \left( \frac{\left( {P25}_{i}-{P10}_{i} \right)+\left( {P50}_{i}-{P25}_{i} \right)+\left( {P75}_{i}-{P50}_{i} \right)+ ({P90}_{i}-{P75}_{i})}{4} \right)$$

Where P10_i_, P25_i_, P50 _i_, P75 _i_ and P90 _i_ correspond respectively to the 10^th^, 25^th^, 50^th^, 75^th^ and 90^th^ percentiles of the amount consumed by participants in the ENNS study of all food items belonging to the serving size subgroup *i*.

In the dietary changes simulation, several modifications, *k*, of the amount consumed of one food item *j* belonging to the food portion subgroup *i,* could be simulated providing that the final simulated quantity would not exceed the 90^th^ percentile of the amount consumed in the serving size subgroup *i,* P90_i_, and be lower than its step (STEP_i,j_). Each final simulated amount consumed by an individual of one food item *j* belonging to the portion size subgroup *i* (Quantity_i_,_j,k,fin_) was calculated from the initial amount consumed of this food item on one eating occasion in the considered diet (Quantity_i,j,ini_) as follows:

$$\mathrm{Quantity}_{i,j,k,fin}=\left( \mathrm{Round}\left( \frac{\mathrm{Quantity}_{i,j,ini}}{\mathrm{STEP}_{i,j}} \right)+ k \right)\times\mathrm{STEP}_{i,j}$$

Finally, after deleting the food items not recommended during pregnancy, and those containing soy (which should only be consumed in small quantities during pregnancy) (39), 1024 food items were considered for type-1 dietary changes.

*Type-2: substitution of a consumed food item with one from the same food subgroup*

In this type, a food item consumed by an individual could be replaced by a food item from the same subgroup. In order to ensure more realistic dietary changes, a food item was selected as a potential substitute providing that it was sufficiently consumed in its subgroup, it was not too expensive by comparison with other items in its subgroup and it was available for sale at least 10 months a year according to different interprofessional federations (for the “Fruits, fresh” subgroup only). Following the selection process described above, and deletion of the “mixed salads” subgroup (because items in this subgroup were insufficiently consumed to identify potential substitutes), 1124 could be substituted in the context of type-2 dietary changes. From these, the 18 food items containing soy were excluded (39), 605 were removed because of their occasional consumption, 13 because of their high prices (based on Kantar World Panel data), and 10 (only fresh fruits) because of their short seasonal availability. As a result, only 478 food items were used for the dietary changes.

*Type-3: substitution of a consumed food item with one from the same group or from a subgroup consumed at the same time*

Under type-3 dietary changes, a consumed food item could be replaced by one from the same group (12 groups). Furthermore, some substitutions between food subgroups not belonging to the same group were authorized when they might be consumed at the same time, with reference to the French cultural meal scheme (*e.g.* for dessert, a food item from the “Dairy” group could be replaced by a food item from the “Fruits and Nuts” group, see **Supplementary Material 5**). Two hundred “average foods” were considered as potential substitutes, based on the 1138 food items that could be replaced under type-3 dietary changes. Due to the heterogeneous substitutions that could be made in this type, the serving size of the food item was taken into account. The serving size of the replacement “average food” was calculated from a ratio based on the mean portion of food items composing the “average food” (replacement item) and the mean serving size of items composing the “average foods” being substituted.
