# Supplementary material for: A clear trade-off exists between the theoretical efficiency and acceptability of dietary changes that improve nutrient adequacy during early pregnancy in French women: Combined data from simulated changes modeling and online assessment survey
Source: PLoS One. 2018 Apr 11;13(4):e0194764. doi: 10.1371/journal.pone.0194764 (PMC5895017; doi:10.1371/journal.pone.0194764)

**S1 Fig.** Diagram presenting possible substitutions depending on the meal between food subgroups belonging to different food groups under type-3 dietary changes. The name of the food group is presented in bold in the first row of each box. The names of the food subgroups belonging to the group are presented from the second row to the last row of the box. Food items belonging to food subgroups whose name is written in italics and underlined could be used as substitutes, but only for lunch and dinner. Food items belonging to food subgroups whose name is written in italics, underlined and followed by an asterisk (*) could be used as substitutes, but only for lunch, dinner and snack. Food items belonging to food subgroups whose name is written in italics could be used as substitutes, but only for breakfast and snack. Food items belonging to food subgroups whose name is written without specific typesetting could be used as substitutes, for all meals. Dietary changes between food subgroups belonging to boxes linked by dotted-dashed arrows could only be made for lunch and dinner. Dietary changes between food subgroups belonging to boxes linked by dashed arrows could only be made for breakfast and snack. Dietary changes between food subgroups belonging to boxes linked by continuous arrows could be made for all meals.


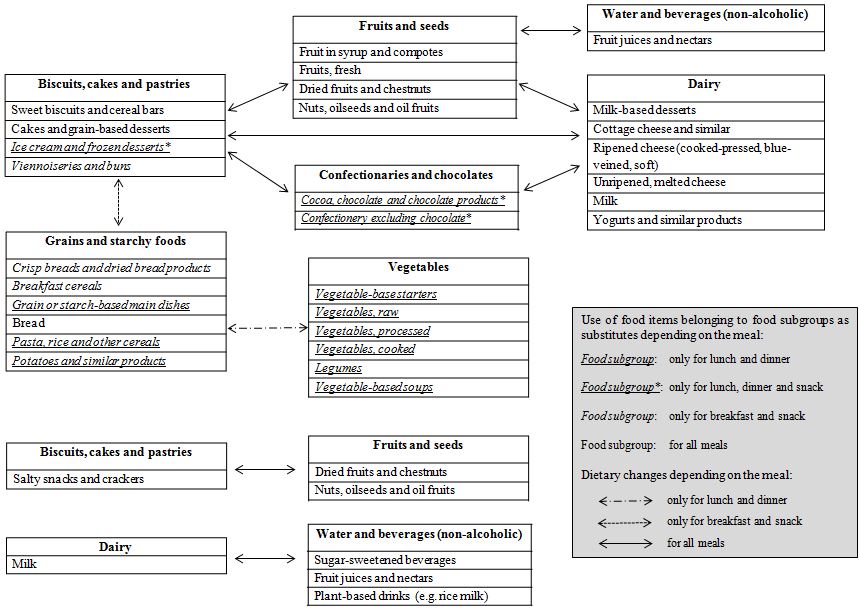

Supplement: S1 Fig — The name of the food group is presented in bold in the first row of each box. The names of the food subgroups belonging to the group are presented from the second row to the last row of the box. Food items belonging to food subgroups whose name is written in italics and underlined could be used as substitutes, but only for lunch and dinner. Food items belonging to food subgroups whose name is written in italics, underlined and followed by an asterisk (*) could be used as substitutes, but only for lunch, dinner and snack. Food items belonging to food subgroups whose name is written in italics could be used as substitutes, but only for breakfast and snack. Food items belonging to food subgroups whose name is written without specific typesetting could be used as substitutes, for all meals. Dietary changes between food subgroups belonging to boxes linked by dotted-dashed arrows could only be made for lunch and dinner. Dietary changes between food subgroups belonging to boxes linked by dashed arrows could only be made for breakfast and snack. Dietary changes between food subgroups belonging to boxes linked by continuous arrows could be made for all meals. (DOCX) [file pone.0194764.s014.docx]
