# Supplementary material for: A clear trade-off exists between the theoretical efficiency and acceptability of dietary changes that improve nutrient adequacy during early pregnancy in French women: Combined data from simulated changes modeling and online assessment survey
Source: PLoS One. 2018 Apr 11;13(4):e0194764. doi: 10.1371/journal.pone.0194764 (PMC5895017; doi:10.1371/journal.pone.0194764)

**S2 Fig.** Diagram of subject flow and reasons for non-inclusions in the study of the acceptability of dietary changes during pregnancy.


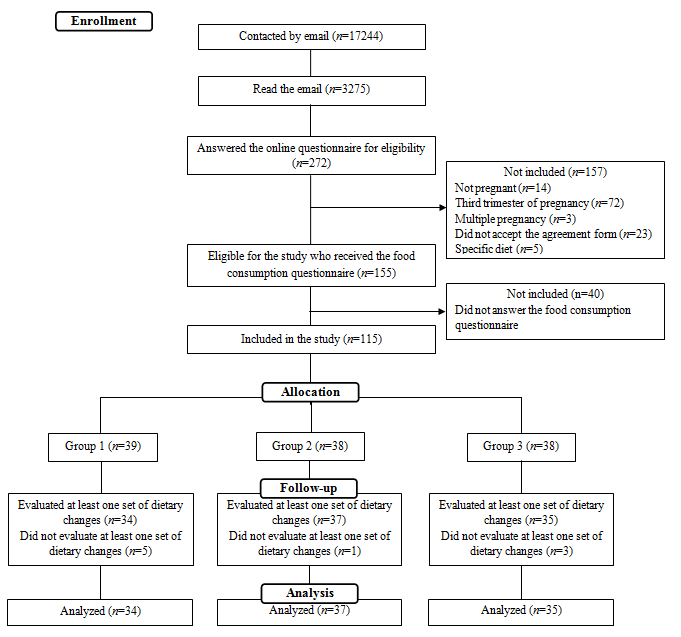

Supplement: S2 Fig — (DOCX) [file pone.0194764.s015.docx]
